# Dysregulation of Complement System and CD4+ T Cell Activation Pathways Implicated in Allergic Response

**DOI:** 10.1371/journal.pone.0074821

**Published:** 2013-10-08

**Authors:** Alexessander Couto Alves, Sören Bruhn, Adaikalavan Ramasamy, Hui Wang, John W. Holloway, Anna-Liisa Hartikainen, Marjo-Riitta Jarvelin, Mikael Benson, David J. Balding, Lachlan J. M. Coin

**Affiliations:** 1 Department of Epidemiology and Biostatistics, Imperial College London, MRC-HPA Centre for Environment and Health, Imperial College London, London, United Kingdom; 2 Department of Medical and Molecular Genetics, King's College London, London, United Kingdom; 3 Genetics Institute, University College London, United Kingdom; 4 Department of Clinical and Experimental Medicine, Linköping University, Linköping, Sweden; 5 Human Development and Health, Faculty of Medicine, University of Southampton, Southampton, United Kingdom; 6 Dept of Paediatrics, Gothenburg University, Gothenburg, Sweden; 7 Institute of Health Sciences, University of Oulu, and Unit of General Practice, University Hospital of Oulu, Oulu, Finland; 8 Biocenter Oulu, University of Oulu, Oulu, Finland; 9 National Institute of Health and Welfare, Oulu, Finland; 10 Department of Clinical Sciences, Obstetrics and Gynecology, Institute of Clinical Medicine, University of Oulu, Oulu, Finland; 11 Department of Genomics of Common Diseases, School of Public Health, Imperial College London, London, United Kingdom; 12 Institute for Molecular Bioscience, University of Queensland, Brisbane, Australia; UTHealth Medical School, United States of America

## Abstract

Allergy is a complex disease that is likely to involve dysregulated CD4+ T cell activation. Here we propose a novel methodology to gain insight into how coordinated behaviour emerges between disease-dysregulated pathways in response to pathophysiological stimuli. Using peripheral blood mononuclear cells of allergic rhinitis patients and controls cultured with and without pollen allergens, we integrate CD4+ T cell gene expression from microarray data and genetic markers of allergic sensitisation from GWAS data at the pathway level using enrichment analysis; implicating the complement system in both cellular and systemic response to pollen allergens. We delineate a novel disease network linking T cell activation to the complement system that is significantly enriched for genes exhibiting correlated gene expression and protein-protein interactions, suggesting a tight biological coordination that is dysregulated in the disease state in response to pollen allergen but not to diluent. This novel disease network has high predictive power for the gene and protein expression of the Th2 cytokine profile (*IL-4, IL-5, IL-10, IL-13*) and of the Th2 master regulator (*GATA3*), suggesting its involvement in the early stages of CD4+ T cell differentiation. Dissection of the complement system gene expression identifies 7 genes specifically associated with atopic response to pollen, including *C1QR1, CFD, CFP, ITGB2*, *ITGAX* and confirms the role of *C3AR1* and *C5AR1.* Two of these genes (*ITGB2* and *C3AR1)* are also implicated in the network linking complement system to T cell activation, which comprises 6 differentially expressed genes. *C3AR1* is also significantly associated with allergic sensitisation in GWAS data.

## Introduction

Asthma, allergic rhinitis and atopic eczema are common allergic diseases with increasing prevalence world-wide. In atopic allergies, allergen induces immunoglobulin E (IgE) formation, which becomes attached to mast cells in a process mediated by CD4+ T cells and known as sensitization [1]. CD4+ T cells are activated by antigen presenting cells (APCs) and differentiate into distinct lineages that are involved in different types of immune responses. In particular, the type 2 T helper (Th2) lineage has a distinct cytokine profile and is associated with allergic reactions. The mechanism by which activated CD4+ T cells are committed to the Th2 lineage is poorly understood, but is thought to involve the dysregulated activation of CD4+ T cells by APCs in allergic individuals [2]–[5]. Therefore, research on the T cell activation pathway is needed to elucidate its interactions with other pathogenic pathways leading to abnormal CD4+ T cell differentiation.

Studying differences in genetic variation between cases and controls, using the genome wide association study (GWAS) design, has led to identification of genetic variation associated with several allergic diseases [6]–[8]. Despite this, it remains challenging to identify the molecular mechanisms underlying allergic disease, and to link these mechanisms with disease pre-disposing genetic variation. Pathway analyses of GWAS have implicated entire pre-defined pathways in disease pathogenesis [9]–[12], but have yet to consider resolving the cell-type associated with the identified pathways and to map these novel pathways on to disease pathophysiology.

One way of mapping GWAS pathway results onto the molecular mechanism of disease is to examine the molecular connections with other disease-dysregulated pathways [13]–[16] and pathway sub-networks [17], [18]. By capitalizing on gene expression studies [2]–[5], GWAS results can be integrated with pathways activated in CD4+ T cells to identify pathogenic pathways at cell level. On the other hand, with the exception of loss-of-function mutations, the observation that complex disease genes have increased tendency for their product to interact and be co-expressed [19], [20] indicates that pathogenic pathways are likely to be significantly linked and coordinated with each other at molecular level. This has support on the observation that disease protein hubs tend to be co-localized in the protein interaction network and enriched for genetic markers of disease [21]. In this context, the integration of protein networks and co-expression networks with genotype data has great potential in identifying dysregulated pathways, and elucidating subnetwork connectivities that have been disrupted in disease.

**Figure 1:**
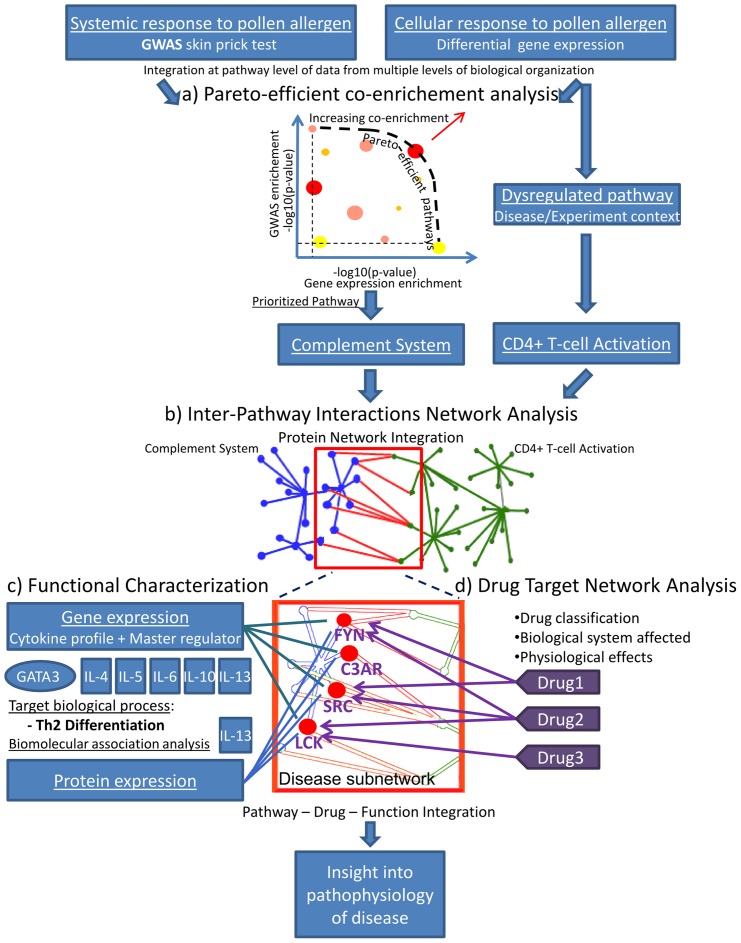
Analysis strategy for identifying coordinated behaviour between disease dysregulated pathways. Disease genes (e.g. FYN, SRC and LCK) that are targeted by anti-inflammatory drugs and associated with biomarkers of disease-relevant biological processes provide insight into the biological function resulting from the coordinated behaviour of both dysregulated pathways identified by integrating GWAS data [6] and gene expression data [2] (a) Co-enrichment analysis of Pareto-efficient pathways identify pathways that are involved in the systemic response to pollen sensitisation and involved in the cellular response to pollen allergen challenge; in this study, complement system was the top hit. (b) Coordination between disease dysregulated pathway (CD4+ T cell activation) and the pathway identified in the disease context (Complement system) is studied using inter-pathway interactions network analysis (INPAR-N). (C) Regression and correlation enrichment analysis is applied to test if the INPAR-N is associated with markers of the biological process involved in the disease onset, i.e. Th2 priming. (D) The genes of the INPAR-N are mapped to disease pathophysiology using drug target network analysis.

We propose a novel methodology to identify pathogenic pathways and we explore the hypothesis that pathways causally linked or involved in disease (e.g. T cell activation in atopy) are interconnected and biologically coordinated, and that this coordination is dysregulated in response to pathophysiological stimuli. We provide a method to select pathways that show a convergent cellular and systemic response to pathophysiological stimuli (Figure 1). We apply this approach to a GWAS on pollen sensitisation, which we integrate with gene expression and supernatant *IL-13* protein levels of CD4+ T cells from allergic individuals and controls cultured with and without allergen. First, we integrate at pathway level gene expression [2] and GWAS data [6] under enrichment analysis; selecting the pathways optimally enriched in both types of data (Pareto-efficient p-values) and ranking them by co-enrichment significance (Figure 1a, see methods). Using this approach, we identify the complement system pathway. Dissection of the complement system gene expression finds 7 genes specifically associated with atopic response. Second, our analysis integrates protein interaction databases with pathway annotation databases using correlation analysis to delineate a molecular network (INter-PAthway inteRactions Network, INPAR-N) between the prioritized pathway (complement system) and the pathways previously implicated in or causally linked to disease (T cell activation pathway) (Figure 1b). Applying this method, we identify a novel network linking the complement system to the T cell activation pathways that is significantly enriched for protein interactions and genes exhibiting correlated expression; suggesting a tight biological coordination. The biological coordination among genes interacting between these pathways is dysregulated in allergic patients but only in response to pollen allergen. We then assess the impact of this inferred network on clinical outcome and thereby identify new susceptibility genes. Third, we study the role of this network on T cell differentiation using gene expression and protein markers of this biological process (Figure 1c). We find that the expression of the genes in the novel disease network have high predictive power for the Th2 cytokine profile (*IL-4, IL-5, IL-10, IL-13*) and for the Th2 master regulator (*GATA3*). Moreover, the expression of the INPAR-N genes is more correlated with these gene sets than the complement system or T cell activation; suggesting its involvement in Th2 priming is probably stronger than its originating pathways. Fourth, since an *in vivo* perturbation analysis of the disease network genes is unfeasible, we map their roles on disease pathophysiology via an *in silico* drug target network analysis (Figure 1d). We assess the reproducibility of the genes differentially expressed and pathways enriched using a replication gene expression dataset that we meta-analyse with our discovery dataset. Our approach suggests that the complement system is likely to modulate atopic CD4+ T cell activation and that the network of genes linking these pathways regulate T cell differentiation, putatively through a mechanism involving T cell danger signal receptors, among other complement receptors; this implies a disruption of the coordination between innate and adaptive immune system in allergic response.

## Materials and Methods

### Gene expression data

Microarray hybridization of discovery and replication samples was conducted using different Illumina microarray chips. Data analysis was conducted on the discovery dataset blind to the replication dataset. In the discovery dataset, gene expression microarray analysis of allergen-challenged CD4+ T cells was performed in samples from 20 Swedish patients with Seasonal Allergic Rhinitis (SAR, see abbreviations and acronyms in Table S1 in Text S1) recruited outside the pollen season (birch pollen-induced allergic rhinitis) according to previously described criteria [22] (analyses using these data have been previously published [21]). Peripheral blood mononuclear cells were cultured in the presence or absence of allergen. IL-13 was measured from the supernatant, and CD4+ T cell were isolated as previously described [2]. The resulting gene expression data after quality control of the assay consists of 13 diluent and 19 allergen stimulated samples (13 paired samples that were analysed as unpaired data).

The replication dataset was obtained from the MultiMod project (http://www.multimod-project.eu/). Microarray analysis was performed in CD4+ T cells in samples from 21 Swedish SAR patients recruited outside the pollen season from Sahlgrenska University hospital in Göteborg, cultured in presence and absence of allergen according to previously described criteria (analyses using these data have been previously published) [2], [23]. The resulting gene expression data of the assay consists of 21 pairs of samples, one diluent and one allergen stimulated for each pair (that were analysed as unpaired data). Gene expression datasets were submitted to GEO with study accession: GSE18574.

### Genome Wide Association Study

The Northern Finland Birth Cohort 1966 (NFBC1966) is a cohort of 12231 individuals born in the provinces of Oulu and Lapland [24]. In 1997 (age 31), 8463 alive were sent postal questionnaires and invited to clinical examination with a 71% response rate (N = 6033). DNA was extracted from blood and 5753 individuals were successfully genotyped using Illumina HumanCNV370-Duo chip. The data was imputed to ∼2.5 million SNPs using NCBI HapMap II CEU build 35 version 21 after pre-filtering SNPs (genotyping call rate >95%, HWE p-value >10^−4^, minor allele frequency >1%) using IMPUTE [25] with confidence call of R^2^>0.5. Genotype and phenotype data were submitted to dbGaP with study accession: phs000276.v1.p1.

Allergic sensitivity to timothy grass pollen was assessed by skin prick test. The longest weal and perpendicular weal were recorded after ten minutes. Participants with a mean weal reaction of at least 3 mm were considered sensitized. Participants with a positive reaction to a negative control (diluent of allergen extracts) or a negative reaction to a positive control (histamine dihydrochloride, 10 mg/mL) were excluded, leaving 693 grass sensitized cases and 3726 controls.

The phenotype-genotype association was conducted using the QUICKTEST software [26]. These analyses were adjusted for sex and relevant principal components determined by a stepwise regression using backward elimination. These data have been previously published as part of a meta-analysis in [6] but have never been analysed separately. This is the first pathway analysis of our GWAS on grass sensitisation.

### Functional annotation analysis and gene set enrichment of gene expression data

Gene set enrichment analysis (GSEA) [9], hypergeometric test-based pathway enrichment analysis [27], and enrichment maps [28] were performed using the R package HTSanaliseR [29]. An enrichment map is a network plot that aids in the identification of mutually overlapping pathways. Functional annotation analysis was performed using DAVID (http://david.abcc.ncifcrf.gov/) [30] setting the filtered microarray gene list as background, and reporting results for category GOTERM_BP_3 with EASE = 0.01 and count = 4. Molecular pathway definitions for the T cell activation process and complement system were downloaded from IPA (Ingenuity® Systems, www.ingenuity.com) and their enrichment for differentially expressed genes was analysed under hypergeometric test and odds ratio analysis. Multiple comparison adjustment used Benjamini-Hochberg q-values method [31] for controlling the false discovery rate (FDR) in both GSEA and Hypergeometric tests as this is the default procedure for DAVID and HTSanaliseR packages.

### Pathway enrichment analysis of genome-wide association study data

To explore potentially new pathways associated with pollen allergic sensitisation in the GWAS dataset, we applied a modified version of the gene set enrichment analysis approach termed meta-analysis gene-set enrichment of variants associations (MAGENTA) [11]. Genes in the genome were mapped to a single SNP with the lowest p-value within a 50 kb upstream or 50 kb downstream window.

### Meta-analysis methodology

Differential gene expression results on discovery and replication dataset were combined via a meta-analysis of the log ratio of means [32]. A gene is considered to be reproducibly differentially expressed [33] if the meta-analysis p-value is smaller than both the p-value obtained in the discovery dataset and the Bonferroni corrected significance level (α = 0.05/42559 = 1.2×10^−6^) Pathway and gene ontology enrichment analysis on discovery and replication datasets were combined using a Mantel-Haenszel meta-analysis of the enrichment odds ratio using the R package rmeta [34]. Fixed effects heterogeneity was assessed by statistical tests setting a cut-off p>0.2 but no test was below this threshold.

### Integration of statistical tests under different null hypothesis for co-enrichment analysis

We propose an approach to establish an association between a series of biological experiments under the constraint that all hypotheses need to be jointly true to corroborate the validity of the conclusion. That is, we need to test if the disjunction of all null hypotheses is false to reject a global null hypothesis. Let *H_k_* denote that the null hypothesis of experiment *k* is true. Then the global null hypothesis *H_g_* is given by:
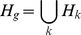



The p-value of the global null hypothesis, which is the chance of any false positives, can be obtained assuming independence by:
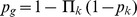



Dropping independence assumption, a conservative estimate can be obtained using the Sidak-corrected *maximum* of the p-values, which is related to the Familywise error rate:
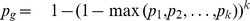



This global p-value is applied to make global inferences from testing different hypothesis in distinct datasets and is used to estimate the co-enrichment and the co-association between GWAS and gene expression results.

### Pathway co-enrichment analysis for data integration from multiple levels of biological organization

We have developed a multi-criteria decision theoretic approach to integrate at pathway-level gene expression and GWAS enrichment analyses. The method selects pathways optimally enriched based on enrichment p-values in both GWAS and gene expression (Pareto efficient pathways) that satisfy nominal significance constraints. A pathway is Pareto efficient if there is no other pathway that optimizes at least one of the objectives (GWAS or gene expression p-value) and is smaller or equal to all other objectives [35]. The outline of the approach is:

1. Select a consensus list of enriched pathways on gene expression data that are simultaneously significant under GSEA and Hypergeometric tests after correction for multiple comparisons.
2. Conduct a GSEA enrichment on full GWAS using MAGENTA.
3. Plot the enrichment p-value for gene expression (the *maximum* of GSEA and Hypergeometric test) against the enrichment p-value of the nominal GSEA test on GWAS using MAGENTA (This plot maps the objective space of Pareto analysis).
4. Identify the pathways that are Pareto-efficient and that are significantly enriched at both levels of biological organization.
5. Integrate GWAS and gene expression significance levels under co-enrichment analysis.
6. Select Pareto-efficient pathways having higher co-enrichment significance and larger effect size based on odds ratio of gene expression enrichment.

### Identification of disease-related genetic subnetworks using inter-pathway interactions (INPAR) analysis

By leveraging systemic properties of disease networks and disease genes, and articulating them with genetic variation and gene expression, we developed a method to identify coordinated behaviour between disease dysregulated pathways and to delineate the corresponding disease network linking these pathways. The method consists of 5 steps:

1. Select a disease dysregulated pathway based on the hypothesis being tested and on the disease context. In this case, one of the fundamental hypotheses underlying the architecture of the experiment is CD4+ T cell activation (after allergen challenge) is dysregulated and involved in abnormal Th2 priming and allergy onset.
2. Identify a pathway associated with disease at cellular and systemic level using co-enrichment of Pareto-efficient pathways.
3. Identify the genes with direct molecular interactions linking both pathways using a curated database (connectivity data from Ingenuity database setting a filter to include only evidence from empirical experiments): the INPAR network (INPAR-N).
4. Test if the INPAR-N network is enriched for direct and indirect protein-protein interactions using DAPPLE [36]. Proteins that are involved in a common mechanism or disease [36]–[38] tend to be highly connected. This step is necessary as the protein interaction graph between pathways tends to be sparsely connected compared to within pathways and thus the INPAR-N might not be densely connected.
5. Test if the INPAR-N network is enriched for genes exhibiting correlated gene expression.
6. Test if the INPAR-N is co-enriched with genetic markers and with genes differentially expressed in response to a disease-associated pathophysiological stimulus (in this case, in response to pollen allergen challenge).

Only direct molecular interactions are selected initially to produce the INPAR-N. In a second stage, an additional layer of molecules that directly interact with the INPAR-N is explored outside the scope of the INPAR-N definition. In this paper, the second stage consists of a bipartite network of genes and drug molecules directly targeting the INPAR-N.

### Regression and gene set correlation enrichment analysis of the Th2 priming biomarkers

To assess the disease network (INPAR-N) gene expression predictive ability for the Th2 priming biomarkers, we have conducted a multiple linear regression analysis of the *IL-13* protein levels and a multivariate regression analysis of the *IL-4, IL-5, IL-6, IL-10, IL-13* gene expression, estimating the predictivity for each model using seven fold cross validation. Statistical significance was assessed using (multivariate regression) Wilks Λ statistic and (multiple regression) Q^2^_y_.

To test the INPAR-N enrichment for genes exhibiting correlated gene expression, we have computed the Spearman correlations among INPAR-N genes and the Spearman correlations between INPAR-N and the remainder genes. We compared the mean absolute correlation of both sets using the Welsh t-test and a permutation test. We assessed the predictive power of the correlation between INPAR-N genes using the area under the curve (AUC) of the Receiver Operating Characteristic (ROC). We extended this concept to assess the co-expression enrichment between pathways. We tested the mean absolute correlations between INPAR-N gene expression and (i) the *IL-13* protein levels, (ii) the Th2 cytokine profile (*IL-4, IL-5, IL-10, IL-13*) and (iii) the Th2 master regulator (*GATA3*). The contrast group was always the remainder genes not including INPAR-N. We repeated this analysis with the gene expression of the complement system and CD4+ T cell activation pathways.

### Cumulative analysis of significant correlation differences between cases and controls for eliciting dysregulated biological coordination within and between pathways

To test the hypothesis of dysregulated biological coordination in response to pathophysiological stimuli, we computed the observed number of nominally significant correlation differences between cases and controls, i.e. 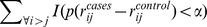. Correlation differences computed within or between gene sets were assessed for statistical significance (*p*) using the R statistical package. The expected number of significant correlation differences was calculated as N × α, where N is the number of correlations tested and α = 0.05. The binomial test with probability of success *P_r_*  = 0.05 was applied to calculate the significance of the difference between observed and expected number of dysregulated correlations, the same calculation based on the t-test was conducted using the disruption rate statistics (#observed significant differences/N) yielding very similar results as expected thus only results based on binomial test are reported.

### Drug target network analysis of the INPAR-N for *in vivo* delineation of gene function and impact on the pathophysiology

The subset of genes that interact between the complement system and T cell activation pathways were assessed for potential drug targets using the IPA database. Analysis of the drug physiological effects was exploited to hint on the function of individual targeted genes in the context of disease states (i.e. via consensus of physiological effects). The integration of the individual physiological contributions enables the mapping of the disease network on the pathophysiology of the disease. Together with the functional annotation characterization of the genes in the context of disease, this analysis can aid the design of new treatments.

### Ethics statement

This research has been approved by the regional ethics committee of the University of Gothenburg and the ethics committee of the Northern Ostrobotnia Hospital District. We obtained written informed consent from all subjects, according to instructions from the ethics committee.

## Results

### Co-enrichment analysis of Pareto-efficient pathways shows that complement system is associated with CD4+ T cell response to pollen allergen challenge and involved in the pathogenesis of pollen allergic sensitisation

**Figure 2:**
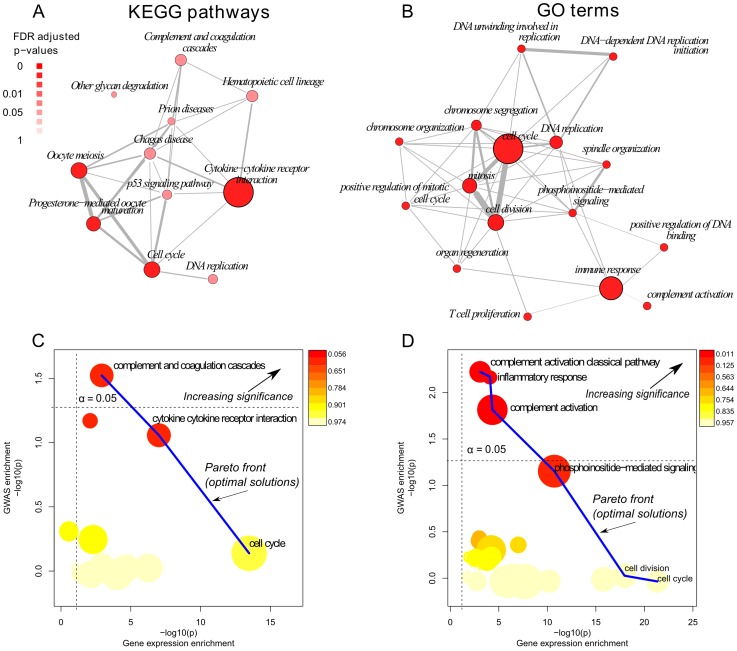
Pareto-efficient co-enrichment integration of gene expression and GWAS data at pathway level. Panel (A) and (B) show the gene expression enrichment maps of the KEGG pathways and GO terms respectively. The degree of gene set overlapping is measured using the Jaccard index and depicted by line thickness. Complement system-related KEGG pathways and GO terms are less overlaped and less likely to be redundant. Panel (C) and (D) depict the KEGG and GO terms plot of objectives for the multicriteria enrichment of gene expression and GWAS data. Pathways are represented as circles with diameter proportional to the odds ratio of the gene expression enrichment and colour coded according to the co-enrichment p-value, pathways closer to the upper right corner are optimally associated at cellular and systemic level with pollen allergen response. Complement system-related pathways and GO terms are Pareto-efficient because they lie on the Pareto front within the significant bounds, suggesting that they may play a role on T cell response to allergen (pollen) and on the pathophysiology of allergic sensitisation with grass pollen.

In order to integrate at pathway level cellular and systemic response to pollen allergen, we combined pathways enriched for differentially expressed genes in atopic CD4+ T cell response to pollen with pathways enriched for genetic markers associated with atopic pollen response to a skin prick test using data from a genome wide association study (Figure 2).

First, we conducted a differential gene expression analysis (Figure S1 showing permutation test and multiple comparison correction). We identified the KEGG pathways and gene ontology (GO) terms enriched using a consensus between GSEA and the Hypergeometric test (Table S2 and Table S3 in Text S1; see Methods S1 in Text S1). Several pathways and biological process categories related to complement system, immunity, cell signalling and cell proliferation were associated with the response to allergen. Enrichment maps analysis showed several mutually overlapping KEGG pathways and GO terms related to cell cycle and cell proliferation, while immune related terms such as T cell proliferation, complement system and immune response are less overlapped and thereby less likely to be redundant (Figure 2, panel A and B).

To identify novel clinically-relevant pathways for pollen allergic sensitisation, we conducted an enrichment analysis under full GWAS of pollen allergic sensitisation using MAGENTA (Table S4 in Text S1). Using annotations from BIOCARTA, Gene Ontology (GO), Ingenuity, Kyoto Encyclopaedia of Genes and Genomes (KEGG), Protein Analysis Through Evolutionary Relationships (PANTHER) databases, we found that ERK/MAPK Signalling, Protein-lipid modification, VEGF Signalling, and Complement System pathways were significantly enriched (α<0.05, FDR <0.25) for SNPs associated with pollen skin prick test responses.

To identify pathways that are clinically relevant for pollen allergic sensitisation and have transcriptional differences that are likely to be causal, versus collateral effects, we identified the pathways optimally enriched in both GWAS and gene expression (with Pareto-efficient p-values) and ranked them by their co-enrichment p-value based on combined analysis of GWAS and gene expression enrichment p-values (see methods) (Figure 2, panel C and D). Complement-system related KEGG pathway and GO terms had convergent cellular and systemic response to pollen allergen challenge. In both annotations, complement system was Pareto-efficient within nominal significance bounds, had larger enrichment odds ratio and higher co-enrichment significance and therefore was prioritized for further analysis.

To confirm that our findings are reliable and reproducible, we have conducted multiple confirmatory and validation analyses (see Methods S1 in Text S1). Briefly, we used a replication gene expression dataset and found that complement system-related KEGG pathways and GO terms were reproducibly enriched in gene expression (KEGG, p = 7.8×10^−7^; GO term, p = 5×10^−12^) (Table S2 and Table S3 in Text S1). To confirm that the complement system enrichment is (i) independent of the annotation database, (ii) independent of the pathway/gene set definition, and (iii) robust to changes on the thresholds of significance to declare genes differentially expressed, we have performed additional functional annotation analyses and replicated these results using DAVID (p = 4.6×10^−3^, Table S5 and Table S6 in Text S1) and Ingenuity Pathway Analysis^TM^ (IPA) definition of the complement system pathway (p = 8.7×10^−7^, Table S7 and Methods S1 in Text S1). Sensitivity analysis of the cut-offs for detecting differentially expressed genes shows that enrichment results of the IPA's complement system was consistent and robust (Methods S1 in Text S1 (statistical sensitivity analysis) and Figure S2). The pathway was significantly enriched on a wide range of FDR cut-offs (0.01<FDR<0.1) even at very low thresholds on effect size (|Log2 r|>0.6; p = 0.03, OR = 2.0). IPA enrichment results were reproduced in the replication dataset (p = 9.2×10^−3^, OR = 3.7) and meta-analysis of the odds ratio increased the significance of the primary result (p = 4.5×10^−11^, OR = 7.2, Table S7 in Text S1). Co-enrichment analysis of the IPA's complement system definition was also significant (p_g_ = 4.6×10^−3^). In the remainder of this paper, we use the IPA definition of the complement system biological process because it is the most comprehensive and specific, as opposed to KEGG that also includes the coagulation cascade and GO terms that focus on partial subsets of the complement system pathway.

To identify individual genes associated with both response to pollen allergen and atopic sensitisation, we have analysed gene expression and GWAS data of the complement system. Interestingly, 16 complement system genes had SNPs significantly (FDR <0.05) associated with pollen allergic sensitisation, including: *C2, C4A, CFB, C6, CFHR3, CFHR1, C3AR1* (Table S8 in Text S1). Meta-analysis of the discovery and replication datasets identified 8 genes differentially expressed at microarray-wide significance level: *C1QBP, C1QR/CD93, C3AR1, C5R1/C5AR1, DF/CFD, ITGAX, ITGB2, PFC/CFP* (Table S9 in Text S1). Remarkably, these genes were all down regulated except *C1QBP*. As the role of *C1QBP, CFD, CFP*, *ITGB2* and *ITGAX* is not yet established in CD4+ T cell allergic response, we conducted an additional experiment comparing the response to pollen in allergic patients and controls. In this experiment, all genes differentially expressed showed consistent direction of effect and most genes were significantly differentially expressed (except *C1QBP*); suggesting they are biomarkers of the dysregulated allergic response (Table 1 and Table S10 in Text S1).

**Table 1:** Complement system and INPAR-N genes differentially expressed in the atopic response to pollen.

	Gene	Discovery dataset		Replication dataset		Meta-analysis	GWAS data		
Pathway	Symbol	P	Log2Ratio	p	Log2Ratio	P	Chr	Pos	Best
									p-val
Complement system	**CFD**	<1E-16	−2.11	<1E-16	−2.67	<1E-16	19	868114	4.40E-02
(CS)	**C5AR1**	9.50E-04	−0.88	<1E-16	−2.03	1.70E-14	19	47770937	6.30E-02
	**CFP**	<1E-16	−1.31	1.00E-05	−0.18	1.50E-12	23	NA*	NA*
	**ITGAX**	2.00E-03	−0.85	7.00E-06	−0.49	9.50E-08	16	31361922	2.20E-02
	**CD93**	5.10E-04	−0.91	1.00E-02	−0.55	1.20E-06	20	23036048	8.90E-02
Overlap CS/INPAR-N	**C3AR1**	9.00E-06	−1.26	3.00E-06	−0.83	3.60E-13	12	8208243	2.00E-03
	**ITGB2**	3.10E-02	−0.51	<1E-16	−1	2.10E-11	21	46313956	3.00E-02
INPAR-N	**FYN**	<1E-16	1.02	4.80E-02	0.29	6.60E-14	6	111999581	3.70E-03
	**STAT4**	1.10E-04	0.58	1.30E-05	0.66	1.20E-13	2	192011105	3.40E-03
	**CD81**	3.80E-04	−0.5	<1E-16	−0.74	1.40E-13	11	2356768	2.30E-01
	**FYN**	<1E-16	−0.95	1.10E-02	−0.28	4.20E-10	6	111999581	3.70E-03
	**ITGAL**	8.20E-03	0.52	3.20E-04	0.35	8.20E-08	16	30518095	1.50E-01
	**FYN**	9.20E-04	0.6	4.90E-03	0.4	1.20E-07	6	111999581	3.70E-03

On GWAS data, genes were mapped to the most significant (best) SNP within a 50Kbp window of the gene. Only complement system genes that are specific markers of atopic response to pollen are shown.

* Not available because only autosomes were analysed. Properdin (CFD) is on the sex chromosome X.

Overall, our results show that the complement system pathway is involved in the atopic sensitisation to pollen, putatively via the atopic response to pollen of CD4+ T cells. We identified 7 genes of the complement system that are specific markers of the atopic response to pollen in CD4+ T cells; including C3AR1, which is also associated with atopic sensitisation to pollen.

### Inter-pathway interactions analysis delineates disease network between complement system and T cell activation that modulate response to pollen allergen challenge

**Figure 3:**
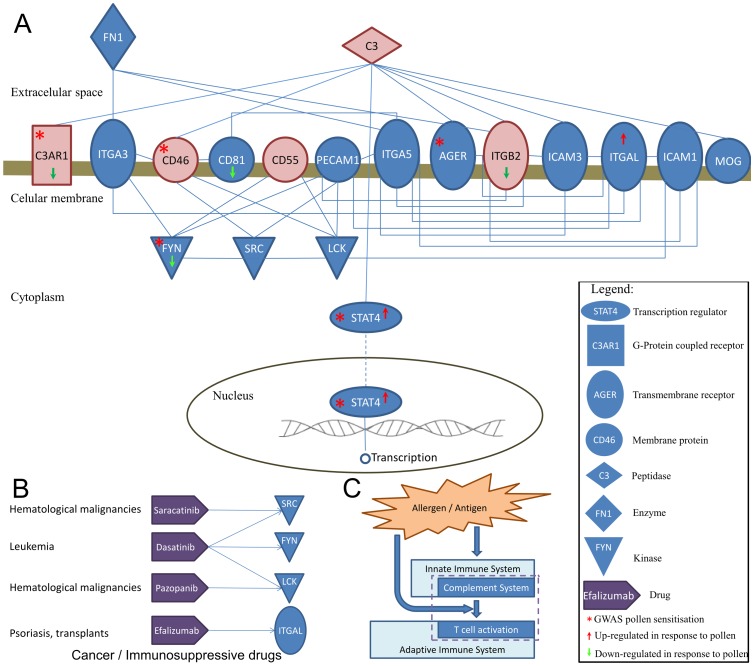
Inter-pathway interactions analysis identifies disease network linking complement system to CD4+ T cell activation. (A) INPAR network has 19 genes that link Complement system to CD4+ T cell activation. Blue nodes correspond to genes involved in T cell activation while red nodes correspond to genes involved in the complement system. (B) Drug target network analysis of the INPAR-N showing that several immunosuppressive drugs target the INPAR-N network genes, particularly the Src family of tyrosine kinases, including Src, Fyn, Lck. (C) Allergens trigger the innate immune system that in turn triggers the adaptive immune system. INPAR-N includes complement system proteins that interact with T cell membrane proteins.

As disease genes tend to cluster together in the protein network neighbourhood [19]–[21], we hypothesize that disease pathways are significantly linked and coordinated with each other. In particular, as dysregulated CD4+ T cell activation is putatively involved in abnormal Th2 differentiation, we hypothesize that the T cell activation pathway is significantly linked and coordinated with the complement system. In order to explore this hypothesis, we conducted an analysis of the protein network between the T cell activation pathway and the complement system and identified a disease network (inter-pathway interactions network, INPAR-N) of 19 genes interacting between both pathways (Figure 3).

To test if the gene products of this disease network are likely to be involved in a common mechanism, we have conducted a protein-protein interaction analysis using DAPPLE [36]. Overall, the INPAR-N is more directly as well as indirectly connected than can be explained by chance despite the fact that the INPAR-N genes were selected from two distinct pathways (Table S11 in Text S1). Individual gene products were also significantly inter-connected in the INPAR-N (α = 0.05) except *MOG, AGER, STAT4*, and *C3AR1*. The gene *C3AR1* is marginally not significant (p = 0.05) because it interacts with 1 protein only, *C3*, which is also part of the INPAR-N (Figure S9). In addition, we found this subnetwork significantly more enriched for genes exhibiting correlated gene expression than complement system or T cell activation alone (Table 2), suggesting a tight biological coordination between these pathways.

**Table 2:** Geneset correlation enrichment analysis shows that INPAR-N is more enriched for genes exhibiting correlated gene expression and is more strongly associated with IL-13 protein levels, Th2 cytokines and Th2 master regulator than T cell activation or complement system.

		Mean absolute correlation			ROC Analysis			
		(Spearman)					95% Confidence Interval	
				p-value				
Pathway	Response Vector	Pathway	Null	t-test	AUC	p-value	lower	Upper
	(data type)			(permutation)				
INPAR-N	IL-13	**0.36**	**0.3**	**0.045**	**0.62**	**0.049**	**0.53**	**0.74**
	(protein levels)			**(0.005)**				
	Th2 cytokine profile	**0.24**	**0.2**	**0.0107**	**0.57**	**0.005**	**0.52**	**0.61**
	(gene expression)			**(0.008)**				
	GATA3	**0.36**	**0.2**	**0.005**	**0.68**	**0.001**	**0.58**	**0.79**
	(gene expression)			**(0.0004)**				
	INPAR-N	**0.31**	**0.2**	**6.22E-09**	**0.59**	**1.71E-07**	**0.55**	**0.62**
	(gene expression)			**(<2.2e-16)**				
Complement system	IL-13	0.3	0.3	0.12	0.55	0.16	0.47	0.56
	(protein levels)			(0.07)				
	Th2 cytokine profile	0.22	0.2	0.05	0.52	0.05	0.49	0.55
	(gene expression)			(0.04)				
	GATA3	0.29	0.2	0.07	0.58	0.07	0.49	0.67
	(gene expression)			(0.04)				
	Complement system	**0.23**	**0.2**	**1.40E-04**	**0.52**	**0.026**	**0.5**	**0.54**
	(gene expression)			**(<2.2e-16)**				
T cell activation	IL-13	**0.33**	**0.3**	**3.40E-11**	**0.62**	**6.10E-12**	**0.59**	**0.66**
	(protein levels)			**(4.00E-15)**				
	Th2 cytokine profile	**0.23**	**0.2**	**4.40E-12**	**0.54**	**7.90E-09**	**0.53**	**0.55**
	(gene expression)			**(<2.2e-16)**				
	GATA3	**0.32**	**0.2**	**6.20E-12**	**0.61**	**7.70E-13**	**0.58**	**0.65**
	(gene expression)			**(<2.2e-16)**				
	T cell activation	**0.26**	**0.2**	**<2.2e-16**	**0.56**	**<2.2e-16**	**0.553**	**0.559**
	(gene expression)			**(<2.2e-16)**				

To test if this network was activated in response to allergen challenge, we conducted an enrichment analysis of the gene expression. The results show a significant enrichment (p = 5.2×10^−3^, OR = 7.6, Table S7 in Text S1). We reproduced these results in a replication dataset and meta-analysis of both datasets increased the significance of the primary analysis (p = 2.3×10^−4^, OR = 5.2; Table S7 in Text S1). We applied the same analysis to the T cell activation pathway and confirmed its involvement in the atopic response to pollen (p_meta_<10^−16^, OR = 7.6; Table S7 in Text S1). To test whether our disease network is clinically relevant for pollen sensitisation and to determine whether the allergen response-associated transcriptional differences observed are likely to be causal, versus collateral effects, we conducted a co-enrichment analysis of the INPAR-N genes. The results show a strong and significant enrichment for SNPs associated with pollen allergic sensitisation (OR = 3.89, p = 6.2×10^−11^) and this association is stronger than that for T cell activation (OR = 1.06, p = 0.29) or complement system (OR = 1.74, p = 2.0×10^−3^) alone.

To identify individual genes associated with both response to pollen allergen and atopic sensitisation, we have analysed gene expression and GWAS data of the disease network. Interestingly, several INPAR-N genes had SNPs significantly associated with pollen allergic sensitisation (Table 1 and Table S12 in Text S1) and were differentially expressed (Table 1 and Table S13 in Text S1). These include: *C3AR1*, *FYN*, and *STAT4* (Figure 3a).

Overall, our results show that the complement system is tightly coordinated with T cell activation, suggesting that both pathways are involved in a common mechanism in allergy. The disease network linking both pathways is associated with pollen sensitisation and with CD4+ T cell response to pollen in allergic patients. The strongest association with allergy in the network linking both pathways is *C3AR1*. The molecular network re-constructed (Figure 3a) from the protein interaction network, gene expression and genetic markers of allergic sensitisation suggest a route linking the adaptive and innate immune system that putatively modulate T cell activation in allergy (Figure 3c).

### Biological coordination between T cell activation and the complement system is dysregulated in allergic individuals response to pollen

In order to explore the hypothesis that dysregulated biological coordination is involved in allergic response to pollen, we analysed the intra and inter-pathway correlations in cases and controls on the replication dataset (as the discovery dataset only has cases). We characterize dysregulated biological coordination as a significant correlation difference between cases and controls (Table 3). In the response to allergen, we have found a significant number of correlation differences within INPAR-N, T cell activation and between T cell activation and the complement system. Interestingly, T cell activation already showed perturbed correlation profile in response to diluent challenge. We also studied the antithetical concept of biological coordination by estimating the mean absolute correlation in cases and controls (Table S13 in Text S1). Consistently, we have found a higher mean absolute correlation in the controls response to pollen when looking at genes in the INPAR-N or T cell activation pathways. Interestingly no differences were found between controls and allergic patients subject to diluent challenge; suggesting that the disruption of the biological coordination was likely caused by the pathophysiological stimulus (pollen challenge).

**Table 3:** Dysregulated biological coordination in response to allergen challenge.

	Allergen			Diluent			
	Correlation differences			Correlation differences			
Pathway	Observed	Expected	p	Observed	Expected	p	N
INPAR-N	**25**	**14**	**5.1E-03**	16	14	4.9E-01	276
Complement System × T-cell Activation	**1054**	**886**	**1.9E-08**	902	886	5.8E-01	17727
Complement System	72	66	4.5E-01	56	66	2.1E-01	1326
T cell activation	**3751**	**2899**	**1.1E-54**	**3423**	**2899**	**2.2E-22**	57970
INPAR-N inter-pathway	11	7	1.8E-01	4	7	3.4E-01	145

The observed and expected number of nominally significant correlation differences between cases and controls are shown for the response to allergen and diluent. Number of correlations denoted by N. Entries in bold denote significantly dysregulated gene sets (α = 0.05). Notice: “Complement System × T-cell Activation” denotes the cross-correlation between the genes of the complement system and the genes of the CD4+ T cell activation pathways.

In order to test if the observed coordination disruption is a consequence of the inactivation of control T cells, we conducted a principal component analysis of the gene expression of cases and controls looking at the following relevant gene sets independently: T cell activation pathway, complement system, INPAR-N as well as the set of genes that were differentially expressed on the discovery dataset in response to pollen (Figure S3). We found, in all gene sets, that controls and cases response to pollen are clustered together and are significantly different from the response to diluent, suggesting that T cells were activated in both cases and controls; this finding is also consistent with literature [39], [40]. We also investigated differences in the CD4+ T cell subtypes (Th1, Th2, Th17 and Treg) by looking at the cytokine profiles of these subtypes and testing for significant differences between cases and controls challenged with and without allergen (Figure S4). We found significant differences in the gene expression of Th2 and to a lesser extent Th17 cytokine profiles, suggesting a response mainly driven by Th2 and Th17 subtypes.

Overall, this result suggests that i) the T cell activation is internally dysregulated, ii) the biological coordination between the complement system and T cell activation pathway is disrupted in response to pollen, and iii) the cytokine profile of the CD4+ T cells is associated with a population of Th2 and Th17 subtypes; suggesting a link between T cell activation dysregulation and CD4+ T cell subtype.

### Disease network highly predictive of Th2 cytokine profile and Th2 master regulator

**Figure 4:**
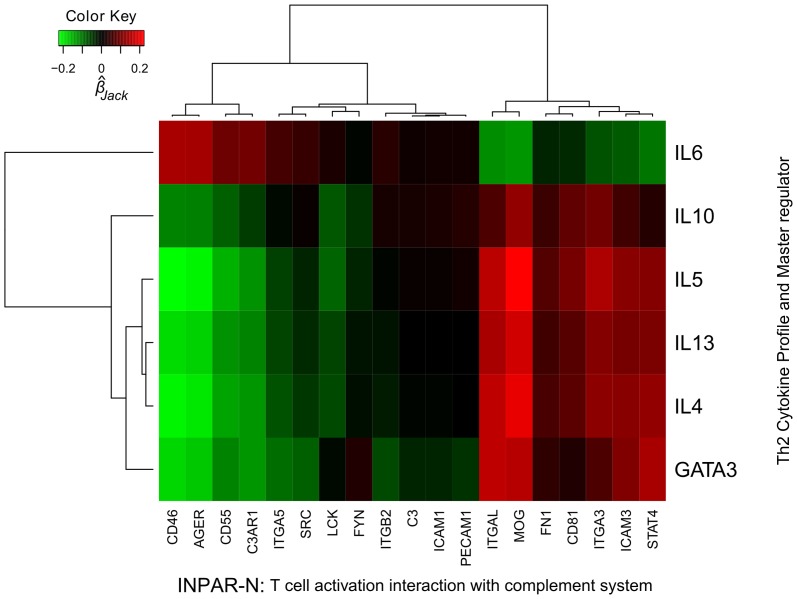
Multivariate regression coefficients of INPAR-N on gene expression data of Th2 cytokine profile and master regulator. The multivariate response vector (y-axis) consists of the Th2 master regulator (GATA3) and the genes involved in the Th2 cytokine profile. The multivariate predictor vector (x-axis) consists of the subset of the gene products linking complement system to CD4+ T cell activation (INPAR-N). Clustering of response and predictor variables is statistically significant (α = 0.05). There is one group of genes that contribute to increase the expression of the Th2 cytokine profile and another group that is down regulating the response. IL-6 behaves differently from all other cytokines, including GATA3. All complement system genes contribute to downregulate the response variables.

To characterize the influence of INPAR-N gene expression on Th2 priming and to test whether its impact on CD4+ T cell differentiation is greater than complement system and T cell activation, we tested three hypotheses. First, we assessed the predictivity of IL-13 protein levels, a marker of Th2 CD4+ T cell subtype, using a regression model of the INPAR-N genes (Table 4 and Figure S8). We found that the INPAR-N gene expression was highly predictive and significantly associated with the supernatant levels of this cytokine (Q^2^_y_ = 0.45, p = 0.001). Spearman correlation analysis found several INPAR-N genes associated with IL-13 protein levels, including *FYN, ITGB2* and *STAT4* that were also differentially expressed in response to pollen (Figure S7). Second, we tested the association of the Th2 cytokine profile and master regulator with INPAR-N genes using a multivariate regression having for response vector *IL-4, IL-5, IL-6, IL-10, IL-13* and *GATA3* (Table 4); results show that the INPAR-N is highly predictive of the gene expression levels of the response vector (out of sample 1-Wilks' lambda = 0.89, p = 0.0002). Analysis of the standardized INPAR-N regression coefficients shows that *GATA3*, *IL-4, IL-5, IL-10, IL-13* are significantly clustered together (bootstrap test alpha<0.05) while the *IL-6* regression coefficient has almost the reverse pattern of association (Figure 4). Interestingly, in our multivariate model all complement system genes contribute to downregulate the Th2 cytokines and *GATA3* expression (Figure 4). We note that all complement genes specifically associated with the atopic response to pollen are also down-regulated. Bi-clustering of the Spearman correlation matrix between INPAR-N and response vector genes reveals that *GATA3* have the strongest association with INPAR-N genes and that *IL-6* and *IL-10* have almost symmetric association patterns comparatively to *IL-4, IL-5, IL-13* and *GATA3* (Figure S5 and Figure S6). Third, we show that the INPAR-N is enriched for genes highly correlated with: i) *IL-13* protein levels, ii) Th2 cytokine profile and iii) Th2 master regulator; both in terms of average absolute correlation and area under the curve (AUC) of the receiver operating characteristic (ROC) (Table 2). Moreover, the INPAR-N correlation enrichment was always larger than complement system and T cell activation (p = 0.016 using binomial test with success probability of 0.5). These results suggest INPAR-N involvement in Th2 priming is probably stronger than its originating pathways and therefore potentially more relevant for the disease pathophysiology. This indicates that the coordinated behaviour between these pathways is likely to play an important role in the allergic response via CD4+ T cell differentiation.

**Table 4:** Regression analysis shows that the disease network gene expression is predictive of the Th2 differentiation markers.

Target	R^2^_y_/1-Wilk's λ	Q^2^_y_/1-Wilk's Λ	Model p-value	Q^2^_y_ p-value
IL-13 protein level	0.73	0.45	3E-04	2E-03
Th-2 cytokine profile	0.92	0.89	1E-11	2E-04

### Drugs targeting the disease network have immune-suppressive and anti-inflammatory effect *in-vivo*

To evaluate the physiological role, delineate gene function and assess whether a molecular intervention on INPAR-N genes can modify the pathophysiology of the disease, we conducted a drug target network analysis. We have queried the Ingenuity database for drugs targeting genes in this network (Table S15 in Text S1) and generated a bipartite network between drugs and genes (Figure 3 panel B). INPAR-N genes are targeted by 5 drugs; 4 of them used in anti-cancer therapy. The consensus physiological response to these drugs consists of an immunosuppressant effect. Among these, *SRC* tyrosine kinase is a target for 3 different drug molecules. Remarkably, the drug molecule dasatinib targets 3 genes of the INPAR-N, including *FYN*. Dasatinib may induce neutropenia along with having potential anti-inflammatory and immunosuppressive effects [41]. It is known that neutrophilia and eosinophilia co-occur in allergen induced airway hyperreactivity (AHR) and are the hallmark of allergic rhinitis and asthma. These network analyses emphasize that multiple genes and their interactions are likely to be involved in the inflammatory response. Conversely, certain genes have a broader impact in the pathophysiology of allergy. *FYN* for example, which is targeted by dasatinib, is highly interconnected with other disease genes in the INPAR-N, is associated with Th2 cytokine profile, is differentially expressed in response to pollen allergen and is associated with atopic sensitisation to pollen.

## Discussion

Previous studies demonstrated that CD4+ T cell counts increase in nasal mucosa of rhinitis patients after allergen challenge [42]. Nasal biopsies of rhinitis patients showed increased *IL-4, IL-5, IL-13* mRNA expression suggesting a Th2 cytokine profile. In our experiment, allergen-challenged PBMCs provided an *in vitro* model of cell-cell interactions among antigen-presenting cells, CD4+ T cells and effector cells in allergic inflammation. In order to understand the mechanisms underlying the response to pollen allergen challenge, CD4+ T cells of SAR patients were isolated and gene expression levels were obtained using Illumina microarrays.

Our co-enrichment analysis of gene expression and genetic variation showed that the complement system was implicated in the systemic response to pollen sensitisation and in the CD4+ T cell response to pollen. These results were reproduced in a replication dataset and meta-analysis of both datasets increased significance of the primary analysis. This suggests that approaches that look at GWAS data at pathway level and integrate GWAS results with gene expression data can uncover new pathways and genes that would not be identified using a routine GWAS or gene expression analysis. Our result confirms the role of the complement system in the immune system homeostasis and disease [43], in the regulation of adaptive immunity [44], and in T cell regulation [45]. Previously, complement system has been associated with allergic rhinitis in gene expression of mucosa biopsies [46]. Multiple complement system proteins have been found to be differentially expressed in the nasal lavage fluids of rhinitis patients, including *C3aR* and *C5aR* [47], and after allergen challenge, *C3a* and *C5a* [48]. The identification of the complement system in the context of T cell activation suggests that complement-associated danger signals (e.g. *C3a* and *C5a*) together with natural allergen may be involved in priming Th2 cells in allergy. This is consistent with previous work that implicates *C5aR* signal transduction in T cell expansion [49] and complement regulator factor H [50] in Th2 allergic inflammatory response in a mouse model. The complement system involvement in other immune disorders is well documented in the literature. *C1, C2, C3, C4* deficiency and increased levels of *C3d* (resulting from the breakdown of *C3*) are associated with Lupus. *C3d* and *C4d* levels are higher in patients with systemic sclerosis; *C1q* and *C2* deficiency as well as increased serum levels of *C3a* and *C5a* have been also related with rheumatoid arthritis [51].

Our gene expression meta-analysis of the complement system pathway shows that both *C3aR* and *C5aR* were downregulated after allergen challenge in patients. In addition, genetic markers in *C3aR* were associated with pollen allergic sensitisation. This is consistent with previous findings that show *in vivo C5aR* targeting during initial allergen exposure can either induce or enhance Th2 adaptive immunity [52] and a knockout asthma mouse model *C5aR* (−/−) exhibits excessive IL17A production that drives a severe asthma phenotype by enhancing Th2 cell-driven pathology [53], [54]. Similarly, knockout mouse model *C3aR* (−/−) exhibits exaggerated Th2 response to epicutaneous sensitisation [55] and allergic asthma [56]. In contrast, pharmacological targeting of *C3, C3a, C5a, C5aR* in an established allergic environment showed a reduction in airway inflammation [52]. Our results extend these results to an *in vitro* human cell model of allergen response showing that complement system plays a role in allergy probably by regulating differentiation of CD4+ T cell towards Th2 subtype or by modulating T cell activation. *C1QR* was down regulated in our study, previously *C1QR1* was found expressed in T cells and when bound to its primary ligand *C1Q* produced an antiproliferative signal, suggesting that it plays a role in T cell activation and proliferation consistent with our observations [57]. *CFP* was down regulated, confirming previous findings that *CFP* regulate the activation of the complement system and is secreted by peripheral blood T cells [58]. We have found complement factor D to be down regulated. Previous work suggested that *CFD* may tilt T cell differentiation towards *IFNG* secreting effector T cells by permitting complement activation during APC/T-cell interactions [59].

Inter-pathway interactions analysis identified a module of genes co-associated and co-enriched with pollen allergic sensitisation and T cell activation. The inferred subnetwork allowed us to identify further disease genes that have smaller differences in expression levels but that may play an important role in disease [60]. The network analysis of direct molecular interactions of the module of genes suggests that the *proto-oncogene tyrosine-protein kinase* gene (*FYN*) together with lymphocyte-specific protein tyrosine kinase (*LCK*) play a central role in the cross-talk between T cell activation and complement system in the allergen response. *FYN* is highly interconnected within this module of genes, it is targeted by anti-inflammatory and immunosuppressive drugs, it is significantly associated with pollen allergic sensitisation, and it is differentially expressed in the allergen challenge response. It also reacts with complement decay accelerating factor *CD55*. *CD55* may suppress T cell response in the context of inflammation [61] and *CD55* deficiency promotes T cell differentiation towards IFNG producing phenotypes like Th1 [59]. *FYN* also interacts directly via *LCK* and *SRC*. *LCK* and *SRC* react with complement regulatory protein *CD46*. The co-stimulation with *CD46* is known to drive the differentiation of CD4+ T cells towards a T-regulatory cell 1 phenotype [62]. T-r1 cells play an active role suppressing activation of bystander T helper cells and acquire a memory phenotype. This suggests that complement system might play a role in allergic response by mediating the differentiation of CD4+ T cells towards Th2 cells via co-stimulation with anaphylatoxins [63]–[65] and impairing differentiation towards the protective T-r1 phenotype due to lower levels of *CD46*. T cells of animals with *FYN* −/− knockout show impaired proliferation and calcium mobilization after TCR stimulation [66]. *FYN* has an active role in the maintenance of anergy state and blocks *IL-2* transcription and therefore T cell proliferation [67]. *FYN* is involved in the mechanism of T cell anergy maintained by a block of the RAS/MAP kinase pathway [68]. Both *Ras* and *MAPK* genes had SNPs significantly associated with pollen allergic sensitisation. Previously, *FYN* was associated with asthma [69]. This suggests that *FYN* might be a key mediator of the complement system role on T cell activation and also on allergic response.

The disease network (INPAR-N) gene expression had strong predictive power of supernatant *IL-13* cytokine protein levels and of Th2 cytokine gene expression profile consisting of *IL-4, IL-5, IL-6, IL-10, IL-13* as well as master regulator of Th2 differentiation *GATA3*. INPAR-N association with these markers of Th2 differentiation was stronger than either complement system or T cell activation alone, suggesting this newly inferred subnetwork could be involved in CD4+ T cell differentiation. This might be corroborated by the stronger than expected connectivity between INPAR-N gene products, as this occurs when genes are involved on a common mechanism. These results are strikingly corroborated by several other studies showing the involvement of multiple INPAR-N genes in Th2 differentiation, e.g. *Lck* expression mediates Th2 differentiation through effect on *GATA3* [70]. Increased expression levels of *CD81* [71] and impairment of *FYN* expression are both implicated in Th2 differentiation [72]. Overall, our study confirms and extends previous work suggesting the importance of complement system for normal T cell differentiation [73] by showing its relevance for allergic sensitisation to pollen and delineating its involvement in CD4+ T cell response to pollen allergen challenge.

This study contributes extensively to the characterization of the gene expression signature of CD4+ T cells allergen response and delineates a putative route for complement system modulation of CD4+ T cell activation. INPAR analysis successfully mapped the interaction between T cell activation and complement system by taking into consideration the interplay among several pathways on coalescence and canalisation of function. Although ultimately only clinical trials in humans can unequivocally show the importance of a given mechanism or intervention strategy in treatment and disease. The methodologies developed in this work can be applied to new problems to perform global inferences from multiple experiments. The integration of data at pathway level using the concept of Pareto-efficiency and co-enrichment analysis can be applied to other diseases to optimally identify the convergent biological functions at the different levels of organization. In this context, pathway level analyses using GSEA and MAGENTA allows the use of all information within a dataset (GWAS or microarray) rather than arbitrary assignment of a significance threshold and concentrating on top hits. The method to identify coordination between dysregulated pathways in disease context could be applied to other problems since the underlying principles such as enrichment for protein interactions, for expression correlations, and for SNPs targeting disease protein hubs, are generalizable to other complex diseases. The application of regression and gene set correlation analysis to markers of the biological processes related to the disease mechanism can also be extended to other conditions to elicit the involvement of disease networks, particularly when multilevel data is available. The proposed drug target analysis is a useful *in silico* method to link gene networks to *in vivo* physiology and pathophysiology. The approach here proposed can bridge translational research in animals and *in vitro* experiments, with human systemic response to disease, allowing to elicit the impact in human physiology long before more complex experiments, usually out of reach of most basic research, are done. Consequentially, these methods have the potential to speeding up the process of identifying good candidate interventions for personalised therapies in other disease areas.

## Supporting Information

Figure S1 **Differential expression analysis of the discovery and replication datasets.** Histograms of the permutation test t-statistics and p-value of the (A) Discovery and (B) Replication dataset. Q-Q plot of the t-statistics in the (C) Discovery and (D) Replication dataset. Correction for multi-comparison using Storey-s q-value false discovery rate showing approach to estimate probability of the null hypothesis as function of the lambda parameter (E) Discovery and (F) Replication datasets. The t-test distributions are symmetrically centred on zero and the p-value histogram is enriched for the bracket of p-values <0.05. The probability of the null hypothesis is greater than 0.6 for both datasets. Microarrays analysis produced no visible artefacts.
(TIF)

Figure S2 **Sensitivity analysis of the complement system enrichment for genes differentially expressed.** Sensitivity of the complement system enrichment as a function of the cut-offs on FDR and gene expression fold change. FDR cut-offs range from 0.01–0.1 while fold change ranges 1–5. (A) Enrichment odds ratio (B) Statististical significance of the enrichment using the one side fisher exact test (C) Number of significant genes withing the complement system pathway.
(TIF)

Figure S3 **CD4+ T cells of cases and controls are activated in response to pollen challenge.** PCA scores plot of the gene expression of (A) T cell activation, (B) Complement system, (C) INPAR-N and (D) Genes differentially expressed in response to pollen in the discovery dataset. MANOVA analysis of the gene expression shows that the differences between cases and controls stimulated with and without pollen is statistically significant for all gene sets.
(TIF)

Figure S4 **Gene expression of the Th2 and Th17 cytokine profiles shows significant differences between cases and controls stimulated with and without pollen.** We applied a MANOVA analysis to the cytokines and master regulator gene expression of cases and controls stimulated with and without pollen. We show for the cytokine profile of each cell subtype the log10 transformed p-value. The dashed lines corresponds to α = 0.05 and α_B_ = 0.05/4 = 0.0125.
(TIF)

Figure S5 **Heat map of the spearman correlation between genes of the Th2 cytokine profile + GATA3 Th2 master regulator with the genes interacting between T cell activation and complement system.**
(TIF)

Figure S6 **Heat map of the multivariate regression coefficient p-values showing the pattern of significant associations between INPAR-N genes and Th2 cytokine profile + GATA3 Th2 master regulator.**
(TIF)

Figure S7 **Spearman's correlation between IL-13 protein level and gene expression of the pathway interaction between** **T cell activation and complement system.**
(TIF)

Figure S8 **IL-13 protein level predictions vs. observations made with regression models fitted using leave one out cross** **validation.**
(TIF)

Figure S9 **INPAR-N with nodes colour coded according to the probability that a protein would be connected to other INPAR-N genes (directly or indirectly) by chance.** The large majority of INPAR-N proteins are highly inter-connected despite being selected from two distinct pathways, suggesting their involvement on common mechanism.
(TIF)

Text S1 **Table S1, Abbreviations and acronyms. Table S2, Meta-analysis of KEGG pathways enrichment showing consensus of GSEA and Hypergeometric test. Complement system is one of the top pathways related to immune system. Table S3, Meta-analysis of the GO term enrichment showing consensus of GSEA and Hypergeometric tests of the gene sets having more than 3% of genes differentially expressed. Table S4, Top 10 pathways associated with allergic grass pollen sensitisation identified by GSEA analysis using MAGENTA. Table S5, Gene ontology terms enrichment analysis using DAVID. Table S6, KEGG pathway enrichment analysis using DAVID. Table S7, Co-enrichment analysis of gene expression and genetic variations using IPA pathway definitions implicates complement system and INPAR-N in the pathogenesis of allergy. Table S8, Complement system genetic variations associated with pollen allergic sensitisation ranked by adjusted p-value. Table S9, Meta-Analysis of the complement system genes differentially expressed in response to pollen in atopic individuals. Table S10, Genes of the complement system differentially expressed between atopic and controls treated with pollen. Table S11, INPAR-N direct and indirect proteins connectivity is statistically significant, suggesting their involvement on common mechanism. Table S12, Disease network INPAR-N genetic variations associated with pollen allergic sensitisation ranked by adjusted p-value. Table S13, Meta-analysis of the disease network (INPAR-N) genes expression showing all transcripts available in both microarrays sorted by meta-analysis p-value. Table S14, Biological coordination in atopic patients (cases) is disrupted in response to pollen allergen (replication dataset). Table S15, Subset of genes that interact between the complement system and T cell activation (Ingenuity® IPA). Methods S1.**
(DOCX)
