# Supplementary figures and images for: Dysregulation of Complement System and CD4+ T Cell Activation Pathways Implicated in Allergic Response
Source: PLoS One. 2013 Oct 8;8(10):e74821. doi: 10.1371/journal.pone.0074821 (PMC3792967; doi:10.1371/journal.pone.0074821)

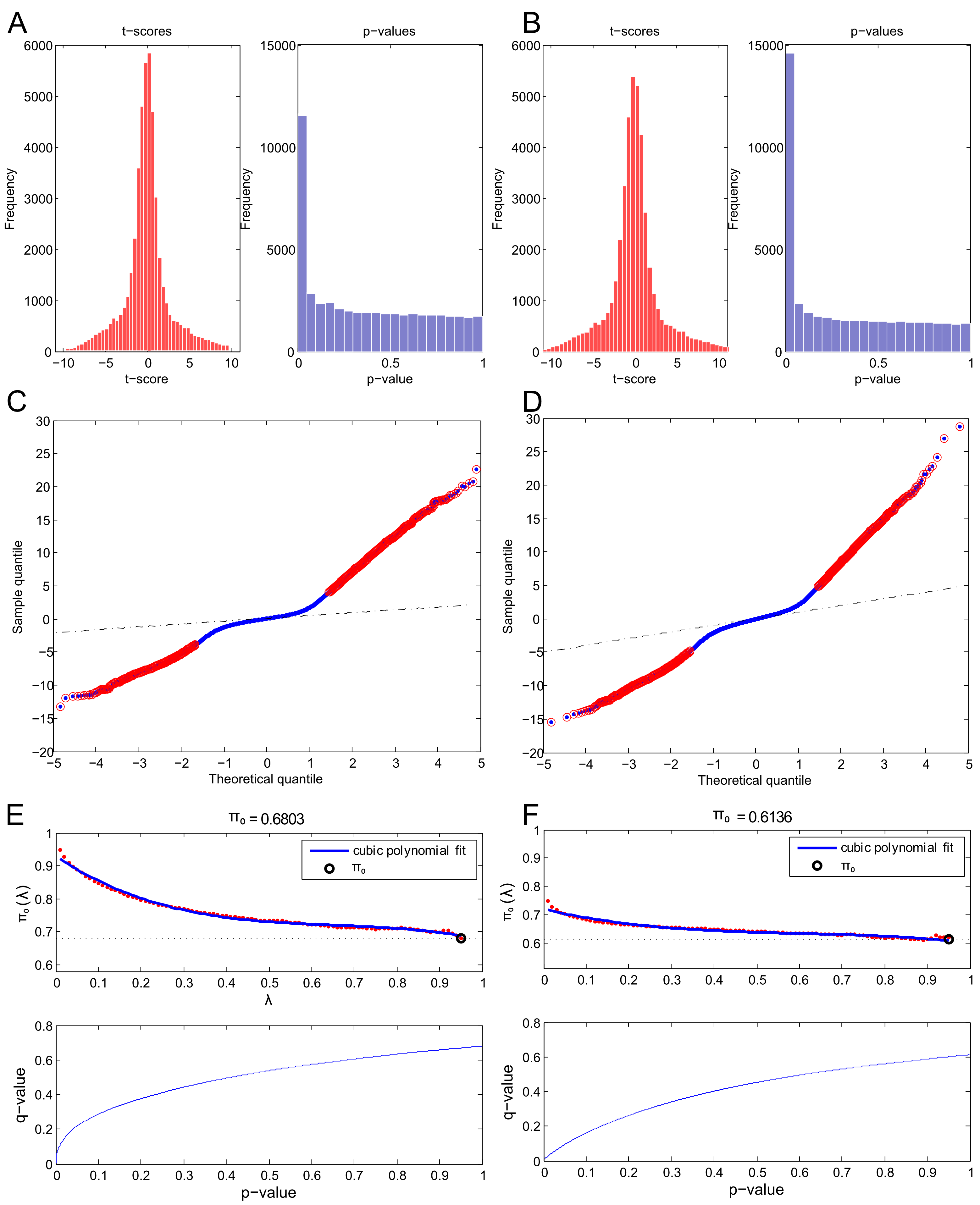

Supplement: Figure S1 — Differential expression analysis of the discovery and replication datasets. Histograms of the permutation test t-statistics and p-value of the (A) Discovery and (B) Replication dataset. Q-Q plot of the t-statistics in the (C) Discovery and (D) Replication dataset. Correction for multi-comparison using Storey-s q-value false discovery rate showing approach to estimate probability of the null hypothesis as function of the lambda parameter (E) Discovery and (F) Replication datasets. The t-test distributions are symmetrically centred on zero and the p-value histogram is enriched for the bracket of p-values <0.05. The probability of the null hypothesis is greater than 0.6 for both datasets. Microarrays analysis produced no visible artefacts. (TIF) [file pone.0074821.s001.tif]

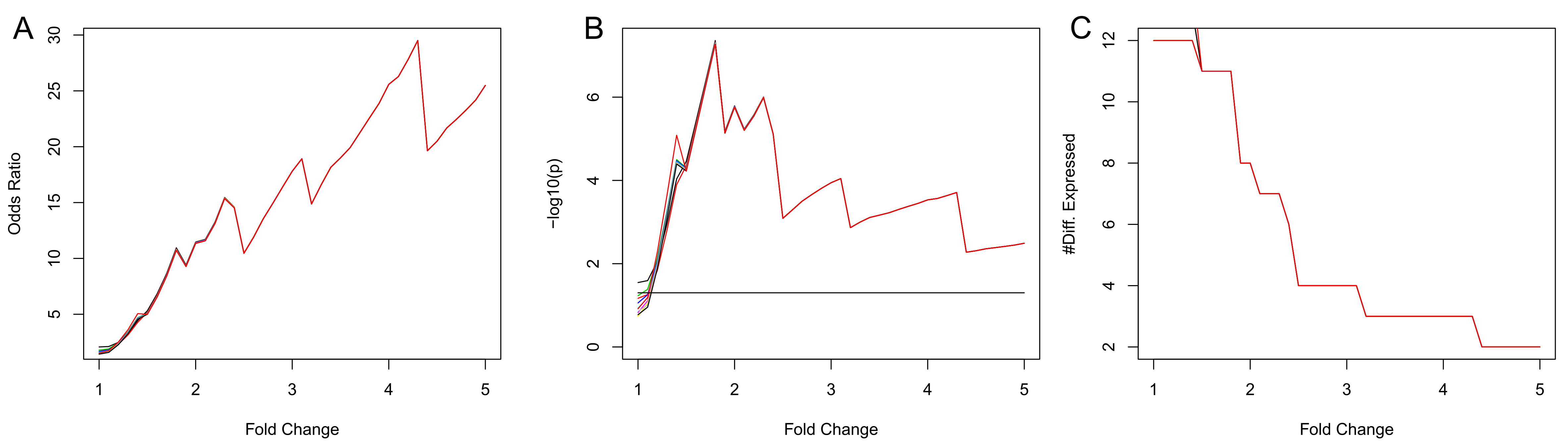

Supplement: Figure S2 — Sensitivity analysis of the complement system enrichment for genes differentially expressed. Sensitivity of the complement system enrichment as a function of the cut-offs on FDR and gene expression fold change. FDR cut-offs range from 0.01–0.1 while fold change ranges 1–5. (A) Enrichment odds ratio (B) Statististical significance of the enrichment using the one side fisher exact test (C) Number of significant genes withing the complement system pathway. (TIF) [file pone.0074821.s002.tif]

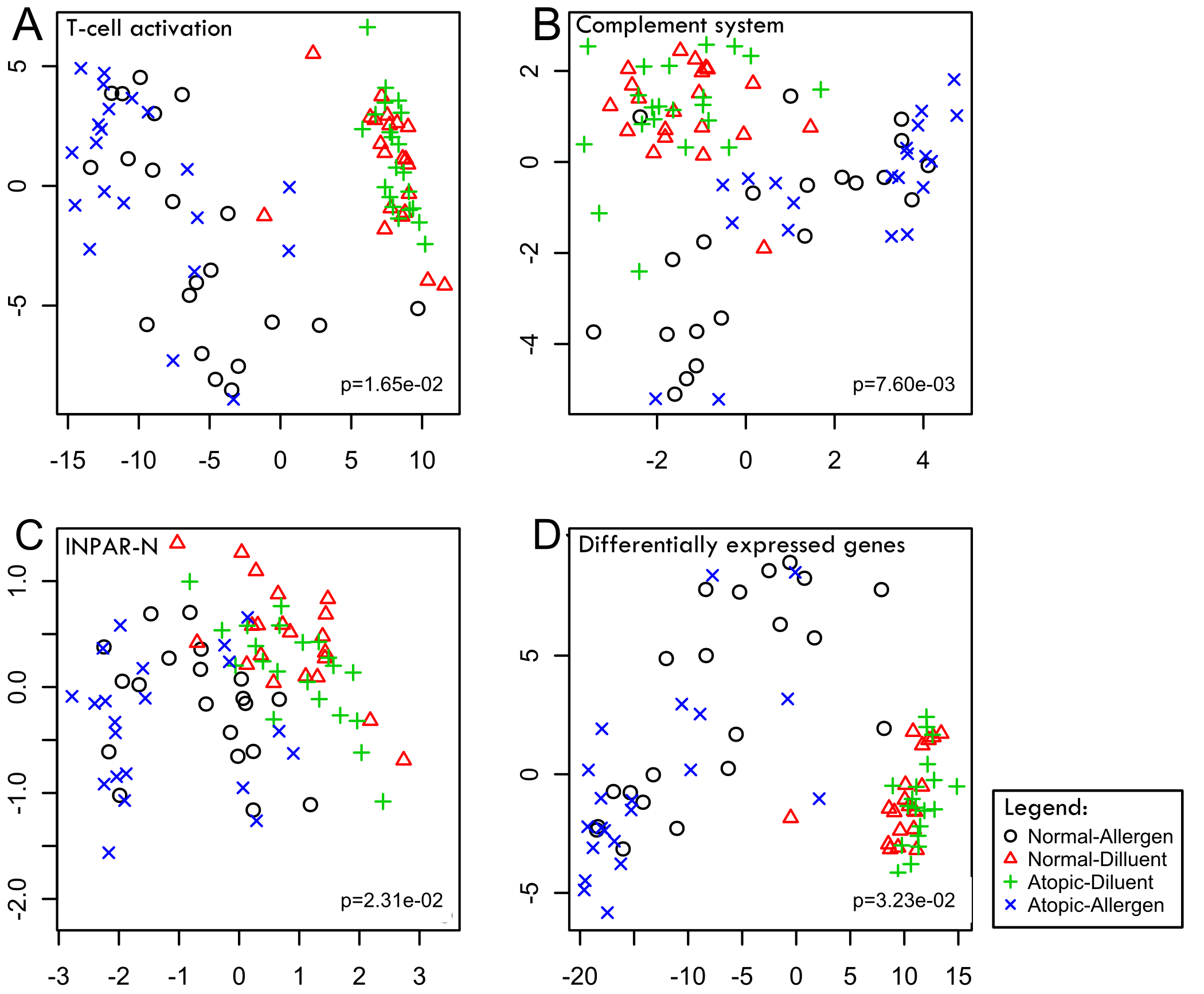

Supplement: Figure S3 — CD4+ T cells of cases and controls are activated in response to pollen challenge. PCA scores plot of the gene expression of (A) T cell activation, (B) Complement system, (C) INPAR-N and (D) Genes differentially expressed in response to pollen in the discovery dataset. MANOVA analysis of the gene expression shows that the differences between cases and controls stimulated with and without pollen is statistically significant for all gene sets. (TIF) [file pone.0074821.s003.tif]

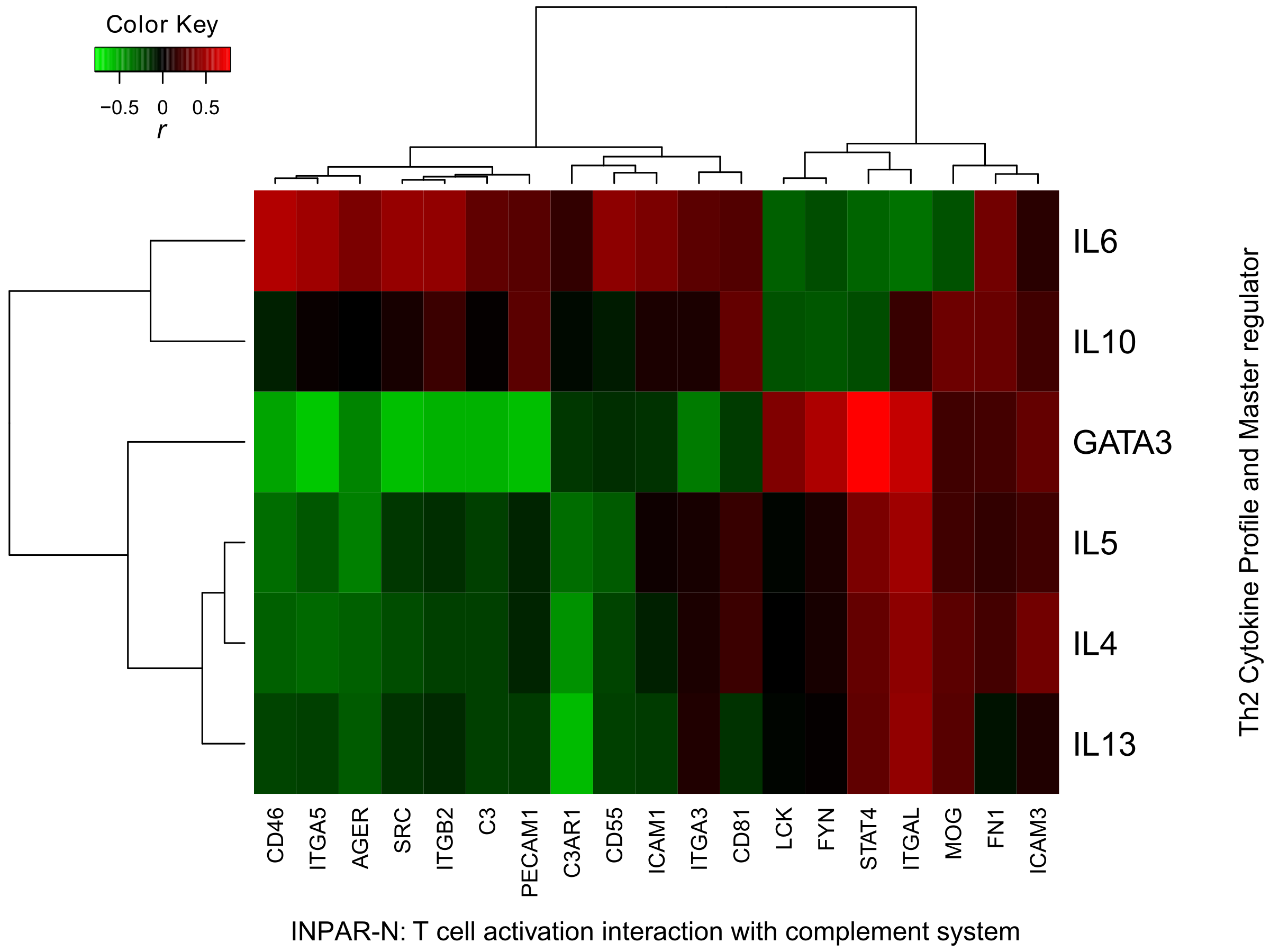

Supplement: Figure S5 — Heat map of the spearman correlation between genes of the Th2 cytokine profile + GATA3 Th2 master regulator with the genes interacting between T cell activation and complement system. (TIF) [file pone.0074821.s005.tif]

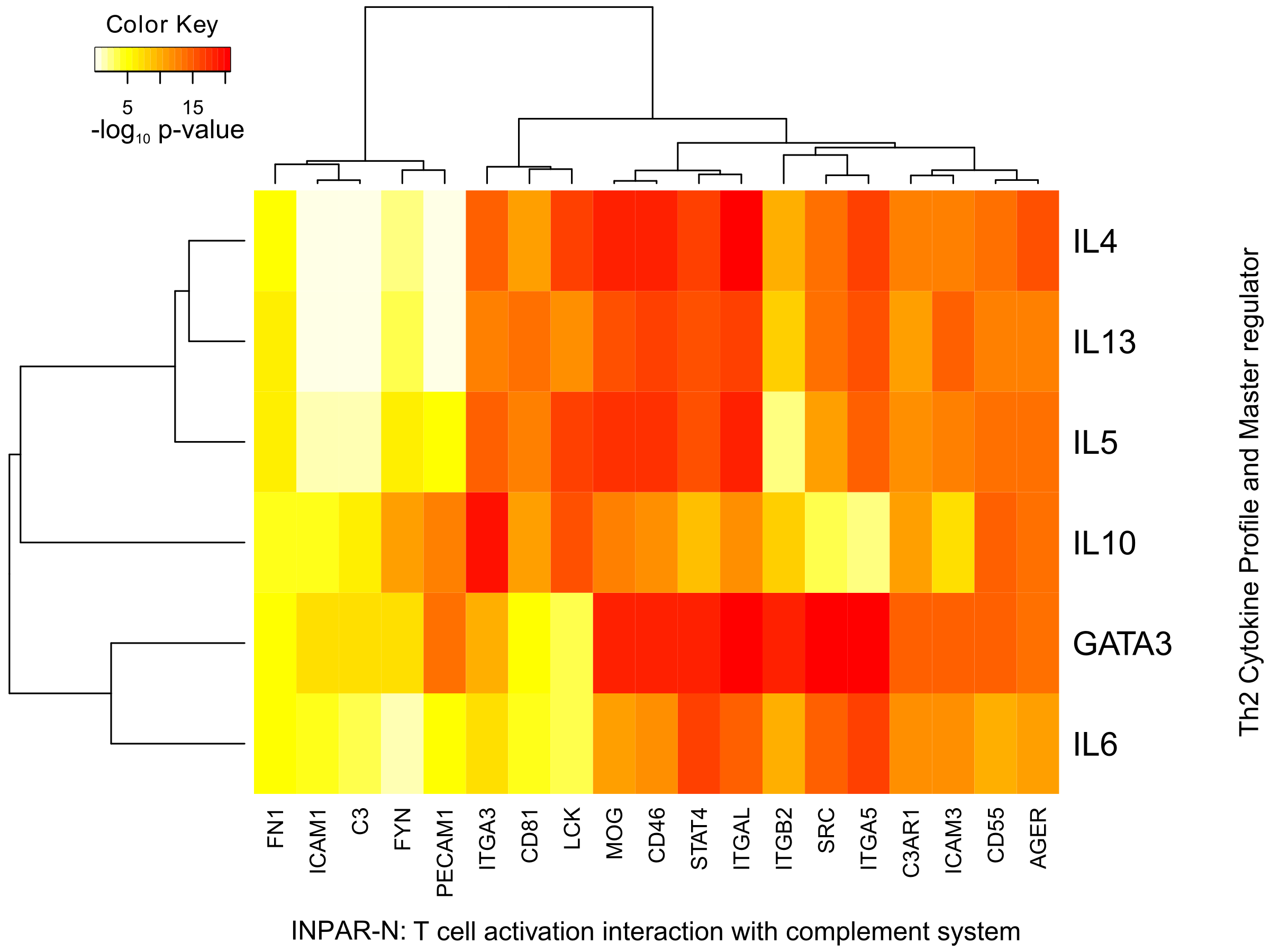

Supplement: Figure S6 — Heat map of the multivariate regression coefficient p-values showing the pattern of significant associations between INPAR-N genes and Th2 cytokine profile + GATA3 Th2 master regulator. (TIF) [file pone.0074821.s006.tif]

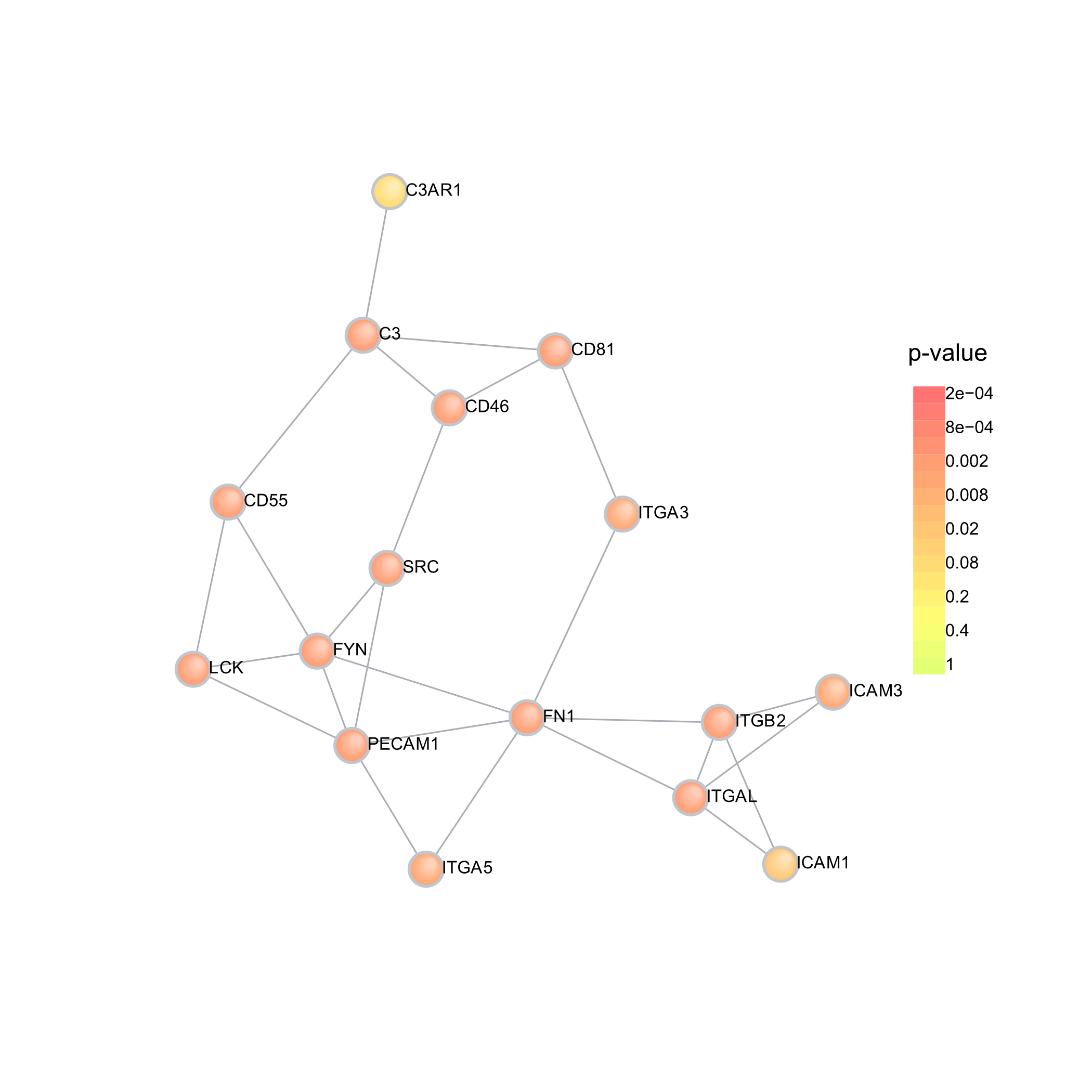

Supplement: Figure S9 — INPAR-N with nodes colour coded according to the probability that a protein would be connected to other INPAR-N genes (directly or indirectly) by chance. The large majority of INPAR-N proteins are highly inter-connected despite being selected from two distinct pathways, suggesting their involvement on common mechanism. (TIF) [file pone.0074821.s009.tif]
