# Supplementary material for: Dysregulation of Complement System and CD4+ T Cell Activation Pathways Implicated in Allergic Response
Source: PLoS One. 2013 Oct 8;8(10):e74821. doi: 10.1371/journal.pone.0074821 (PMC3792967; doi:10.1371/journal.pone.0074821)
Supplement: Text S1 — Table S1, Abbreviations and acronyms. Table S2, Meta-analysis of KEGG pathways enrichment showing consensus of GSEA and Hypergeometric test. Complement system is one of the top pathways related to immune system. Table S3, Meta-analysis of the GO term enrichment showing consensus of GSEA and Hypergeometric tests of the gene sets having more than 3% of genes differentially expressed. Table S4, Top 10 pathways associated with allergic grass pollen sensitisation identified by GSEA analysis using MAGENTA. Table S5, Gene ontology terms enrichment analysis using DAVID. Table S6, KEGG pathway enrichment analysis using DAVID. Table S7, Co-enrichment analysis of gene expression and genetic variations using IPA pathway definitions implicates complement system and INPAR-N in the pathogenesis of allergy. Table S8, Complement system genetic variations associated with pollen allergic sensitisation ranked by adjusted p-value. Table S9, Meta-Analysis of the complement system genes differentially expressed in response to pollen in atopic individuals. Table S10, Genes of the complement system differentially expressed between atopic and controls treated with pollen. Table S11, INPAR-N direct and indirect proteins connectivity is statistically significant, suggesting their involvement on common mechanism. Table S12, Disease network INPAR-N genetic variations associated with pollen allergic sensitisation ranked by adjusted p-value. Table S13, Meta-analysis of the disease network (INPAR-N) genes expression showing all transcripts available in both microarrays sorted by meta-analysis p-value. Table S14, Biological coordination in atopic patients (cases) is disrupted in response to pollen allergen (replication dataset). Table S15, Subset of genes that interact between the complement system and T cell activation (Ingenuity® IPA). Methods S1. (DOCX) [file pone.0074821.s010.docx]

# SUPPLEMENTARY INFORMATION

**Disruption of complement system and CD4+ T cell activation pathways implicated in Allergic response**

**Authors**

Alexessander Couto Alves^1^, Sören Bruhn^4^, Adaikalavan Ramasamy^1,2^, Hui Wang^4,6^, John W. Holloway^5^, Anna-Liisa Hartikainen^10^, Marjo-Riitta Jarvelin^1,7,8,9^, Mikael Benson^4^, David Balding^1,3^, Lachlan Coin^1^

**Table of Contents**

SUPPLEMENTARY TABLES 2

SUPPLEMENTARY METHODS 18

Pathway enrichment for genetic variation associated with clinical phenotypes 18

Genetic variations associated with pollen allergic sensitisation within INPAR-N genes 18

Statistical sensitivity analysis of the complement system enrichment 18

Gene expression microarray pre-processing 19

Differential Expression Analysis 19

REFERENCES 19

# Supplementary Tables

Table S1. Abbreviations and acronyms

| Abbreviation | Description |
| --- | --- |
| APC | Antigen Presenting Cell |
| AUC | Area Under The Curve |
| DAPPLE | Disease Association Protein-Protein Link Evaluator. |
| FDR | False Discovery Rate |
| FWER | Family-wise Error Rate |
| GO | Gene Ontology |
| GSEA | Gene Set Enrichment Analysis |
| GWAS | Genome wide association study |
| INPAR-N | INter-PAthway inteRactions Network |
| KEGG | Kyoto Encyclopaedia of Genes and Genomes |
| MAGENTA | Meta-Analysis Gene-set Enrichment of variaNT Associations |
| NFBC1966 | Northern Finland Birth Cohort 1966 |
| OR | Odds Ratio |
| PANTHER | Protein Analysis Through Evolutionary Relationships |
| ROC | Receiver Operating Characteristic |
| SAR | Seasonal Allergic Rhinitis |

Table S2.Meta-analysis of KEGG pathways enrichment showing consensus of GSEA and Hypergeometric test. Complement system is one of the top pathways related to immune system

|  | Discovery | | | | | | Replication | | | | | | Meta-Analysis | | |
| --- | --- | --- | --- | --- | --- | --- | --- | --- | --- | --- | --- | --- | --- | --- | --- |
| Summary | GSEA | Hypergeometric | | | | | GSEA | Hypergeometric | | | | | Hypergeometric | |  |
| **term** | **q-value** | **%** | **count** | **OR** | **p** | **q-value** | **q-value** | **%** | **count** | **OR** | **p** | **q-value** | OR | p | p-het |
| Cell cycle | <5E-4 | 10.7 | 13 | 21.9 | 2.2E-14 | 1.4E-11 | <5E-4 | 12.9 | 16 | 16.6 | 3.3E-15 | 1.5E-12 | 18.6 | <1E-16 | 0.50 |
| DNA replication | <5E-4 | 8.6 | 3 | 15.5 | 6.2E-05 | 4.2E-03 | <5E-4 | 17.1 | 6 | 22.1 | 3.5E-08 | 5.9E-06 | 19.3 | <1E-16 | 0.64 |
| Oocyte meiosis | <5E-4 | 6.5 | 7 | 11.9 | 3.4E-07 | 8.3E-05 | <5E-4 | 6.3 | 7 | 7.2 | 1.2E-05 | 6.2E-04 | 9.0 | <1E-16 | 0.37 |
| Progesterone-mediated oocyte maturation | <5E-4 | 6.0 | 5 | 10.6 | 1.3E-05 | 1.3E-03 | <5E-4 | 5.9 | 5 | 6.6 | 1.7E-04 | 5.6E-03 | 8.2 | 4.1E-14 | 0.47 |
| Cytokine-cytokine receptor interaction | <5E-4 | 4.3 | 11 | 7.9 | 6.3E-08 | 2.1E-05 | <5E-4 | 8.9 | 23 | 11.4 | 4.6E-17 | 3.1E-14 | 9.9 | <1E-16 | 0.36 |
| p53signaling pathway | <5E-4 | 4.5 | 3 | 7.7 | 7.8E-04 | 1.6E-02 | <5E-4 | 5.9 | 4 | 6.6 | 4.9E-04 | 9.8E-03 | 7.0 | 7.1E-09 | 0.84 |
| **Complement and coagulation cascades** | <5E-4 | 4.5 | 3 | 7.7 | 7.8E-04 | 1.6E-02 | 3.9E-02 | 4.7 | 3 | 5.2 | 3.3E-03 | 3.2E-02 | 6.2 | 7.8E-07 | 0.63 |
| Chagas disease | 8.8E-02 | 3.0 | 3 | 5.1 | 3.4E-03 | 4.5E-02 | <5E-4 | 3.9 | 4 | 4.2 | 3.1E-03 | 3.1E-02 | 4.6 | 1.8E-05 | 0.82 |
| T cell receptor signaling pathway | <5E-4 | 2.8 | 3 | 4.7 | 4.4E-03 | 5.4E-02 | <5E-4 | 4.8 | 5 | 5.3 | 5.2E-04 | 9.8E-03 | 5.1 | 7.7E-07 | 0.89 |
| Chemokine signaling pathway | 4.6E-02 | 2.2 | 4 | 3.7 | 5.3E-03 | 6.3E-02 | <5E-4 | 6.5 | 12 | 7.6 | 2.8E-08 | 5.3E-06 | 6.0 | 6.9E-15 | 0.23 |
| Pyrimidine metabolism | <5E-4 | 2.1 | 2 | 3.5 | 2.0E-02 | 7.8E-02 | <5E-4 | 4.3 | 4 | 4.7 | 2.1E-03 | 2.3E-02 | 4.2 | 1.8E-04 | 0.75 |
| Jak-STAT signaling pathway | 3.5E-02 | 2.0 | 3 | 3.3 | 1.5E-02 | 7.8E-02 | <5E-4 | 4.7 | 7 | 5.2 | 1.0E-04 | 3.8E-03 | 4.4 | 6.0E-07 | 0.52 |
| Ubiquitin mediated proteolysis | 5.6E-02 | 1.5 | 2 | 2.5 | 4.8E-02 | 8.3E-02 | 6.3E-02 | 1.5 | 2 | 1.6 | 1.4E-01 | 1.7E-01 | 1.9 | 1.8E-01 | 0.65 |

Table S3. Meta-analysis of the GO term enrichment showing consensus of GSEA and Hypergeometric tests of the gene sets having more than 3% of genes differentially expressed. Complement system is among the top GO terms related to the immune system.

|  | Discovery | | | | | | Replication | | | | | | Meta-Analysis | | |
| --- | --- | --- | --- | --- | --- | --- | --- | --- | --- | --- | --- | --- | --- | --- | --- |
| Summary | GSEA | Hypergeometric | | | | | GSEA | Hypergeometric | | | | | Hypergeometric | | |
| **Term** | **q-value** | **%** | **count** | **OR** | **p** | **q-value** | **q-value** | **%** | **count** | **OR** | **p** | **q-value** | **OR** | **p** | p-het |
| spindle organization | <5E-4 | 28.6 | 4 | 67.6 | 1.6E-08 | 5.9E-06 | <5E-4 | 42.9 | 6 | 81.5 | 2.1E-11 | 6.3E-09 | 75.3 | <1E-16 | 8.2E-01 |
| chromosome organization | <5E-4 | 23.1 | 3 | 50.2 | 9.4E-07 | 1.7E-04 | 3.9E-02 | 20.0 | 3 | 26.7 | 1.0E-05 | 5.7E-04 | 34.9 | <1E-16 | 4.9E-01 |
| phosphoinositide-mediated signaling | <5E-4 | 21.9 | 7 | 48.6 | 1.5E-11 | 8.0E-09 | <5E-4 | 28.1 | 9 | 43.1 | 2.8E-13 | 1.1E-10 | 45.4 | <1E-16 | 8.4E-01 |
| DNA-dependent DNA replication initiation | <5E-4 | 20.0 | 3 | 41.8 | 1.8E-06 | 2.9E-04 | <5E-4 | 25.0 | 4 | 35.8 | 3.1E-07 | 4.8E-05 | 38.2 | <1E-16 | 8.6E-01 |
| **Complement activation** | <5E-4 | 18.2 | 2 | 36.8 | 3.6E-05 | 3.0E-03 | 2.5E-01 | 9.1 | 1 | 10.6 | 4.7E-03 | 4.1E-02 | 20.2 | 5.8E-12 | 3.3E-01 |
| mitotic chromosome condensation | <5E-4 | 16.7 | 2 | 33.2 | 4.8E-05 | 3.5E-03 | <5E-4 | 25.0 | 3 | 35.7 | 3.8E-06 | 2.8E-04 | 34.6 | <1E-16 | 9.4E-01 |
| chromosome segregation | <5E-4 | 11.6 | 5 | 22.4 | 2.4E-07 | 6.5E-05 | <5E-4 | 11.1 | 5 | 13.5 | 4.2E-06 | 3.0E-04 | 16.8 | <1E-16 | 4.5E-01 |
| Mitosis | <5E-4 | 8.9 | 17 | 18.5 | 1.3E-16 | 1.1E-13 | <5E-4 | 10.5 | 21 | 13.6 | 3.0E-17 | 2.7E-14 | 15.4 | <1E-16 | 4.0E-01 |
| cell division | <5E-4 | 8.5 | 20 | 18.1 | 8.8E-19 | 1.2E-15 | <5E-4 | 10.8 | 27 | 14.5 | 5.8E-22 | 7.8E-19 | 15.8 | <1E-16 | 5.1E-01 |
| DNA replication | <5E-4 | 8.0 | 11 | 15.5 | 5.2E-11 | 2.3E-08 | <5E-4 | 10.4 | 15 | 13.1 | 6.3E-13 | 2.1E-10 | 14.0 | <1E-16 | 7.0E-01 |
| regulation of cell cycle | <5E-4 | 7.1 | 3 | 12.8 | 1.3E-04 | 7.5E-03 | 2.9E-02 | 2.3 | 1 | 2.5 | 6.4E-02 | 1.1E-01 | 6.3 | 3.7E-05 | 1.4E-01 |
| anaphase-promoting complex-dependent proteasomal ubiquitin-dependent protein catabolic process | <5E-4 | 6.3 | 4 | 11.4 | 4.3E-05 | 3.5E-03 | <5E-4 | 6.5 | 4 | 7.4 | 3.2E-04 | 9.1E-03 | 9.0 | 6.9E-13 | 5.6E-01 |
| positive regulation of ubiquitin-protein ligase activity involved in mitotic cell cycle | <5E-4 | 6.3 | 4 | 11.2 | 4.7E-05 | 3.5E-03 | <5E-4 | 4.8 | 3 | 5.3 | 3.1E-03 | 3.1E-02 | 7.6 | 1.1E-09 | 3.4E-01 |
| regulation of cyclin-dependent protein kinase activity | <5E-4 | 5.6 | 2 | 9.7 | 1.4E-03 | 2.5E-02 | 1.7E-02 | 8.1 | 3 | 9.4 | 4.2E-04 | 9.8E-03 | 9.5 | 3.4E-09 | 9.7E-01 |
| cell cycle | <5E-4 | 6.4 | 28 | 9.7 | 3.7E-22 | 9.8E-19 | <5E-4 | 8.2 | 38 | 11.1 | 1.1E-25 | 3.0E-22 | 11.1 | <1E-16 | 8.6E-01 |
| receptor-mediated endocytosis | <5E-4 | 5.0 | 2 | 8.7 | 1.9E-03 | 3.1E-02 | <5E-4 | 2.6 | 1 | 2.8 | 5.4E-02 | 1.1E-01 | 5.1 | 2.1E-03 | 3.5E-01 |
| microtubule-based movement | <5E-4 | 4.5 | 3 | 7.8 | 7.8E-04 | 1.6E-02 | <5E-4 | 7.5 | 6 | 8.8 | 1.2E-05 | 6.2E-04 | 8.4 | 2.7E-13 | 8.8E-01 |
| immune response | <5E-4 | 3.8 | 12 | 7.0 | 7.8E-08 | 2.3E-05 | <5E-4 | 7.5 | 24 | 9.5 | 6.2E-16 | 3.3E-13 | 8.4 | <1E-16 | 4.2E-01 |
| innate immune response | <5E-4 | 3.4 | 4 | 5.9 | 8.2E-04 | 1.7E-02 | 1.1E-01 | 2.7 | 3 | 2.9 | 2.3E-02 | 1.1E-01 | 4.1 | 8.2E-05 | 3.7E-01 |
| negative regulation of ubiquitin-protein ligase activity involved in mitotic cell cycle | <5E-4 | 3.3 | 2 | 5.6 | 6.4E-03 | 7.3E-02 | <5E-4 | 5.0 | 3 | 5.6 | 2.6E-03 | 2.7E-02 | 5.6 | 2.5E-05 | 1.0E+00 |
| inflammatory response | <5E-4 | 3.2 | 7 | 5.6 | 6.6E-05 | 4.4E-03 | 2.0E-01 | 6.2 | 14 | 7.4 | 4.8E-09 | 1.1E-06 | 6.7 | <1E-16 | 5.7E-01 |
| nucleosome assembly | <5E-4 | 3.1 | 2 | 5.3 | 7.3E-03 | 7.8E-02 | 9.9E-02 | 5.7 | 4 | 6.5 | 5.6E-04 | 1.0E-02 | 6.0 | 1.2E-06 | 8.2E-01 |

Table S4. Top 10 pathways associated with allergic grass pollen sensitisation identified by GSEA analysis using MAGENTA. Complement releated pathways shown in bold.

|  |  |  | 95th percentile cutoff | | | | 75th percentile cutoff | | | |
| --- | --- | --- | --- | --- | --- | --- | --- | --- | --- | --- |
| **Database** | **Gene Set** | **#Genes in category tested by GSEA** | **Nominal GSEA**  **p-value** | **FDR corrected**  **p-value** | **Expected #genes above cutoff** | **Observed #genes above cutoff** | **Nominal GSEA**  **p-value** | **FDR corrected**  **p-value** | **Expected #genes above cutoff** | **Observed #genes above cutoff** |
| Ingenuity | ERK MAPK Signalling | 22 | 0.0056 | 0.07 | 1 | 5 | 0.481 | 1 | 6 | 6 |
| PANTHER biological process | Protein-lipid modification | 18 | 0.0016 | 0.08 | 1 | 5 | 0.279 | 1 | 5 | 6 |
| Ingenuity | VEGF Signalling | 15 | 0.0059 | 0.14 | 1 | 4 | 0.056 | 0.79 | 4 | 7 |
| Ingenuity | **Complement System** | 22 | 0.0201 | 0.25 | 1 | 4 | 0.156 | 1 | 6 | 8 |
| PANTHER biological process | Acyl-CoA metabolism | 17 | 0.047 | 0.55 | 1 | 3 | 0.106 | 0.89 | 4 | 7 |
| PANTHER biological process | rRNA metabolism | 59 | 0.0264 | 0.62 | 3 | 7 | 0.023 | 0.91 | 15 | 22 |
| Panther | Insulin/IGF pathway-mitogen activated protein kinase /MAP kinase cascade | 19 | 0.0638 | 0.67 | 1 | 3 | 0.334 | 0.97 | 5 | 6 |
| PANTHER molecular function | Exoribonuclease | 25 | 0.0338 | 0.67 | 1 | 4 | 0.001 | 0.09 | 6 | 14 |
| KEGG | **Complement and coagulation cascades** | 60 | 0.0282 | 0.70 | 3 | 7 | 0.319 | 1 | 15 | 17 |
| GO term | heme biosynthetic process | 15 | 0.0328 | 0.71 | 1 | 3 | 0.145 | 0.94 | 4 | 6 |

Table S5.Gene ontology terms enrichment analysis using DAVID. Significant results (q<0.05) are sorted by fold enrichment.

| DAVID GO_BP_3 Annotation | Discovery dataset | | | | |
| --- | --- | --- | --- | --- | --- |
| Name | Count | % | P | Fold  Enrichment | q-value |
| Cytokinesis | 4 | 3.4 | 4.3E-03 | 12.1 | 5.8E-02 |
| **Complement activation** | 4 | 3.4 | 4.6E-03 | 11.8 | 6.2E-02 |
| organelle fission | 20 | 16.9 | 2.0E-14 | 10.8 | 2.8E-13 |
| humoral immune response | 6 | 5.1 | 4.3E-04 | 9.4 | 5.9E-03 |
| mitotic cell cycle | 22 | 18.6 | 1.3E-12 | 7.3 | 1.8E-11 |
| cell cycle phase | 23 | 19.5 | 1.3E-12 | 6.9 | 1.8E-11 |
| microtubule cytoskeleton organization | 8 | 6.8 | 1.8E-04 | 6.7 | 2.5E-03 |
| regulation of cell cycle | 15 | 12.7 | 3.9E-07 | 5.6 | 5.4E-06 |
| cell cycle process | 24 | 20.3 | 8.8E-11 | 5.2 | 1.2E-09 |
| regulation of immune response | 8 | 6.8 | 2.3E-03 | 4.4 | 3.1E-02 |
| defense response | 19 | 16.1 | 1.7E-06 | 3.8 | 2.4E-05 |
| negative regulation of developmental process | 8 | 6.8 | 4.8E-03 | 3.8 | 6.5E-02 |
| regulation of immune system process | 11 | 9.3 | 1.0E-03 | 3.5 | 1.4E-02 |
| response to wounding | 15 | 12.7 | 8.4E-05 | 3.5 | 1.2E-03 |
| negative regulation of molecular function | 9 | 7.6 | 5.4E-03 | 3.3 | 7.3E-02 |
| cytoskeleton organization | 11 | 9.3 | 2.6E-03 | 3.1 | 3.6E-02 |
| regulation of response to stimulus | 11 | 9.3 | 4.1E-03 | 2.9 | 5.6E-02 |
| negative regulation of cellular process | 34 | 28.8 | 4.0E-07 | 2.5 | 5.6E-06 |
| regulation of cell proliferation | 16 | 13.6 | 1.5E-03 | 2.5 | 2.1E-02 |
| negative regulation of biological process | 35 | 29.7 | 9.8E-07 | 2.4 | 1.4E-05 |
| regulation of catalytic activity | 16 | 13.6 | 3.0E-03 | 2.3 | 4.1E-02 |
| regulation of multicellular organismal process | 17 | 14.4 | 3.2E-03 | 2.2 | 4.3E-02 |
| positive regulation of biological process | 29 | 24.6 | 2.3E-03 | 1.8 | 3.2E-02 |
| regulation of cellular process | 71 | 60.2 | 2.0E-03 | 1.3 | 2.7E-02 |

Table S6. KEGG pathway enrichment analysis using DAVID

|  | Count | % | P-value | FDR | Fold Enrichment |
| --- | --- | --- | --- | --- | --- |
| Cell cycle | 12 | 10.2 | 3.3E-08 | 3.3E-07 | 9.2 |
| Progesterone-mediated oocyte maturation | 5 | 4.2 | 1.1E-02 | 1.1E-01 | 5.6 |
| **Complement and coagulation cascades** | 4 | 3.4 | 3.3E-02 | 2.9E-01 | 5.6 |
| Oocyte meiosis | 6 | 5.1 | 5.0E-03 | 4.9E-02 | 5.2 |
| Cytokine-cytokine receptor interaction | 11 | 9.3 | 2.6E-04 | 2.6E-03 | 4.0 |

Table S7.Co-enrichment analysis of gene expression and genetic variations using IPA pathway definitions implicates complement system and INPAR-N in the pathogenesis of allergy. Meta-analysis of molecular pathways enrichment for gene differenially expressed. Molecular pathway enrichment for genetic variation associated with clinical phenotypes using p-value <10^-2^ cut-off for SNPs within a conservative 5Kbp window around the 3' and 5' region of the gene.

|  | Gene expression enrichment | | | | | | Genetic variation enrichment | | Co-enrichment |
| --- | --- | --- | --- | --- | --- | --- | --- | --- | --- |
|  | Discovery | | Replication | | Meta-Analysis | | NFBC 1966 | | (FWER) |
| Pathway | OR | p-value | OR | p-value | OR | p-value | OR | p-value | p-value |
| **Complement System** | 11.5 | 8.7×10^-7^ | 3.7 | 9.2×10^-3^ | 7.2 | 4.5×10^-11^ | 1.74 | 2.0×10^-3^ | 4.6×10^-3^ |
| T cell activation | 8.8 | <10^-16^ | 7.6 | <10^-16^ | 8.1 | <10^-16^ | 1.06 | 2.9×10^-1^ | 0.5 |
| **INPAR-N** | 7.6 | 5.2×10^-3^ | 3.1 | 9.0×10^-2^ | 5.2 | 2.3×10^-4^ | 3.89 | 6.2×10^-11^ | 4.7×10^-4^ |

Table S8. Complement system genetic variations associated with pollen allergic sensitisation ranked by adjusted p-value. The gene association p-value was computed with MAGENTA algorithm using SNPs within a window of 50Kbp around the gene. The best SNP with minimum p-value is reported with the corresponding FDR. Genes denoted in bold are significantly associated with pollen sensitisation (FDR < 0.05). The correlation between the best SNP p-value and # SNPs/Gene or #independent SNPs/Gene was not significant (p>0.32, r=-0.13 and r=-0.15 respectively).

|  | Gene | | | | | | Best SNP | |  |
| --- | --- | --- | --- | --- | --- | --- | --- | --- | --- |
| Symbol | adjusted | Chr | start | end | #SNPs/Gene | #Independ | Pos | p | FDR |
|  | p-value |  |  |  |  | SNPs/Gene |  |  |  |
| **C2** | 2.03E-05 | 6 | 31868775 | 31913449 | 112 | 14 | 31821040 | 1.20E-05 | 5.40E-04 |
| **C6** | 3.16E-02 | 5 | 41142335 | 41261540 | 263 | 34 | 41142605 | 6.67E-04 | 1.50E-02 |
| **CFB** | 2.44E-02 | 6 | 31913720 | 31919861 | 78 | 11 | 31915613 | 1.68E-03 | 1.80E-02 |
| **C4A** | 2.44E-02 | 6 | 31949833 | 32003194 | 94 | 14 | 31915613 | 1.68E-03 | 1.80E-02 |
| **C3AR1** | 3.90E-02 | 12 | 8210918 | 8218955 | 57 | 11 | 8208243 | 1.96E-03 | 1.80E-02 |
| **CFHR3** | 3.16E-02 | 1 | 196743929 | 196763203 | 45 | 5 | 196771124 | 3.59E-03 | 2.10E-02 |
| **CFHR1** | 3.78E-02 | 1 | 196788860 | 196801319 | 61 | 6 | 196771124 | 3.59E-03 | 2.10E-02 |
| **C9** | 1.20E-01 | 5 | 39284377 | 39364655 | 155 | 20 | 39314746 | 4.13E-03 | 2.10E-02 |
| **CLU** | 2.08E-01 | 8 | 27454450 | 27472327 | 141 | 21 | 27482869 | 4.26E-03 | 2.10E-02 |
| **CD46** | 1.30E-01 | 1 | 207925382 | 207968861 | 73 | 13 | 207980900 | 7.14E-03 | 3.00E-02 |
| **C5** | 1.41E-01 | 9 | 123714613 | 123812554 | 167 | 14 | 123835198 | 7.27E-03 | 3.00E-02 |
| **CR1L** | 2.01E-01 | 1 | 207818457 | 207897036 | 127 | 14 | 207923080 | 9.64E-03 | 3.60E-02 |
| **ITGAM** | 1.88E-01 | 16 | 31271287 | 31344213 | 71 | 11 | 31264266 | 1.14E-02 | 3.90E-02 |
| **C1QBP** | 3.83E-01 | 17 | 5336098 | 5342471 | 108 | 13 | 5308307 | 1.65E-02 | 4.80E-02 |
| **C1QA** | 3.92E-01 | 1 | 22963117 | 22966175 | 99 | 13 | 22924417 | 1.72E-02 | 4.80E-02 |
| **C1QC** | 3.75E-01 | 1 | 22970117 | 22974603 | 100 | 14 | 22924417 | 1.72E-02 | 4.80E-02 |
| ITGAX | 3.45E-01 | 16 | 31366508 | 31394318 | 62 | 13 | 31361922 | 2.22E-02 | 5.60E-02 |
| C4BPA | 4.34E-01 | 1 | 207277606 | 207318317 | 103 | 12 | 207273956 | 2.35E-02 | 5.60E-02 |
| C4BPB | 4.70E-01 | 1 | 207262211 | 207273337 | 82 | 12 | 207273956 | 2.35E-02 | 5.60E-02 |
| ITGB2 | 5.47E-01 | 21 | 46305867 | 46348753 | 134 | 19 | 46313956 | 2.98E-02 | 6.20E-02 |
| C1RL | 3.15E-01 | 12 | 7247145 | 7261869 | 52 | 6 | 7306842 | 3.04E-02 | 6.20E-02 |
| C8B | 6.07E-01 | 1 | 57394882 | 57431688 | 156 | 18 | 57469960 | 3.15E-02 | 6.20E-02 |
| C1QB | 5.17E-01 | 1 | 22979681 | 22988029 | 91 | 11 | 22952385 | 3.28E-02 | 6.20E-02 |
| CFI | 4.93E-01 | 4 | 110661847 | 110723335 | 93 | 16 | 110612536 | 3.33E-02 | 6.20E-02 |
| CFD | 5.01E-01 | 19 | 859664 | 863610 | 36 | 10 | 868114 | 4.36E-02 | 7.70E-02 |
| VTN | 5.03E-01 | 17 | 26694298 | 26697373 | 52 | 8 | 26725264 | 4.44E-02 | 7.70E-02 |
| C1S | 4.14E-01 | 12 | 7167979 | 7178335 | 28 | 7 | 7120395 | 4.62E-02 | 7.70E-02 |
| C1R | 5.16E-01 | 12 | 7187514 | 7245043 | 67 | 11 | 7162597 | 5.28E-02 | 8.50E-02 |
| C1QL2 | 7.68E-01 | 2 | 119913818 | 119916471 | 120 | 14 | 119957656 | 5.61E-02 | 8.70E-02 |
| C3 | 8.03E-01 | 19 | 6677845 | 6720662 | 121 | 30 | 6691630 | 6.01E-02 | 9.00E-02 |
| C5AR1 | 6.62E-01 | 19 | 47813103 | 47825327 | 47 | 10 | 47770937 | 6.32E-02 | 9.00E-02 |
| C1QL1 | 7.16E-01 | 17 | 43037060 | 43045644 | 72 | 14 | 42993854 | 6.38E-02 | 9.00E-02 |
| C1QL3 | 8.08E-01 | 10 | 16555741 | 16564004 | 116 | 18 | 16581628 | 6.74E-02 | 9.10E-02 |
| C7 | 8.03E-01 | 5 | 40909598 | 40983042 | 214 | 20 | 40991343 | 6.87E-02 | 9.10E-02 |
| C8A | 8.46E-01 | 1 | 57320442 | 57383894 | 141 | 28 | 57364040 | 7.16E-02 | 9.10E-02 |
| CD59 | 8.69E-01 | 11 | 33724555 | 33758025 | 171 | 27 | 33731544 | 7.29E-02 | 9.10E-02 |
| CR2 | 7.77E-01 | 1 | 207627644 | 207663240 | 106 | 11 | 207668413 | 8.63E-02 | 1.00E-01 |
| CR1 | 7.82E-01 | 1 | 207669472 | 207815110 | 165 | 15 | 207668413 | 8.63E-02 | 1.00E-01 |
| CD93 | 8.66E-01 | 20 | 23059992 | 23066977 | 153 | 12 | 23036048 | 8.88E-02 | 1.00E-01 |
| CFH | 8.37E-01 | 1 | 196621007 | 196716634 | 121 | 10 | 196580038 | 1.37E-01 | 1.50E-01 |
| C8G | 8.24E-01 | 9 | 139839697 | 139841426 | 48 | 4 | 139860630 | 1.46E-01 | 1.60E-01 |
| CD55 | 8.64E-01 | 1 | 207494816 | 207534311 | 80 | 6 | 207563312 | 1.74E-01 | 1.90E-01 |
| CFHR5 | 9.32E-01 | 1 | 196946666 | 196978803 | 83 | 12 | 197008364 | 2.19E-01 | 2.30E-01 |
| CFHR2 | 9.83E-01 | 1 | 196912933 | 196928356 | 66 | 13 | 196939189 | 3.67E-01 | 3.80E-01 |
| CFHR4 | 9.74E-01 | 1 | 196857143 | 196887843 | 82 | 10 | 196867232 | 3.75E-01 | 3.80E-01 |
| CFP | NA | 23 | 47483611 | 47489704 | NA | NA | NA | NA |  |

Table S9. Meta-Analysis of the complement system genes differentially expressed in response to pollen in atopic individuals. Gene denoted in bold are reproducubly differentially expressed at microarray-wide significance level α<1.2×10^-6^. Trasnscripts available in both microarrays are sorted by meta-analysis p-value

|  | Discovery dataset | | | | Replication dataset | | | | Meta-analysis |
| --- | --- | --- | --- | --- | --- | --- | --- | --- | --- |
| Accession | Symbol | FDR | p | Log2Ratio | Symbol | FDR | p | Log2Ratio | p |
| mRNA RefSeq |  |  |  |  |  |  |  |  |  |
| NM_001212 | **C1QBP** | 9.00E-06 | <1E-16 | 1.01 | C1QBP | 2.00E-06 | <1E-16 | 1.01 | <1E-16 |
| NM_001928 | **DF** | <1E-16 | <1E-16 | -2.11 | CFD | <1E-16 | <1E-16 | -2.67 | <1E-16 |
| NM_001736 | **C5R1** | 5.00E-03 | 9.50E-04 | -0.88 | C5AR1 | <1E-16 | <1E-16 | -2.03 | 1.70E-14 |
| NM_004054 | **C3AR1** | 9.70E-05 | 9.00E-06 | -1.26 | C3AR1 | 1.80E-05 | 3.00E-06 | -0.83 | 3.60E-13 |
| NM_002621 | **PFC** | 1.00E-06 | <1E-16 | -1.31 | CFP | 4.90E-05 | 1.00E-05 | -0.18 | 1.50E-12 |
| NM_000211 | **ITGB2** | 9.10E-02 | 3.10E-02 | -0.51 | ITGB2 | <1E-16 | <1E-16 | -1 | 2.10E-11 |
| NM_000887 | **ITGAX** | 9.60E-03 | 2.00E-03 | -0.85 | ITGAX | 3.40E-05 | 7.00E-06 | -0.49 | 9.50E-08 |
| NM_012072 | **C1QR1** | 3.00E-03 | 5.10E-04 | -0.91 | CD93 | 2.40E-02 | 1.00E-02 | -0.55 | 1.20E-06 |
| NM_001734 | C1S | 6.00E-01 | 7.00E-01 | 0.03 | C1S | 1.00E-06 | <1E-16 | 0.81 | 1.50E-05 |
| NM_000063 | C2 | 7.90E-04 | 1.10E-04 | -1.31 | C2 | 7.80E-03 | 2.90E-03 | -0.47 | 1.10E-04 |
| NM_001710 | BF | 1.70E-01 | 7.50E-02 | 0.23 | CFB | 7.70E-04 | 2.20E-04 | 0.78 | 1.10E-04 |
| NM_000573 | CR1 | 6.30E-01 | 8.10E-01 | -0.01 | CR1 | 7.00E-06 | 1.00E-06 | -0.3 | 1.50E-04 |
| NM_001877 | CR2 | 6.60E-01 | 9.30E-01 | 0 | CR2 | 1.00E-04 | 2.40E-05 | -0.34 | 8.10E-03 |
| NM_000632 | ITGAM | 6.50E-01 | 9.30E-01 | 0.17 | ITGAM | 1.50E-05 | 3.00E-06 | -1.11 | 1.70E-02 |
| NM_172369 | C1QG | 2.00E-06 | <1E-16 | -2.46 | C1QC | 5.30E-01 | 7.00E-01 | 0.82 | 2.80E-02 |
| NM_000491 | C1QB | 4.00E-05 | 3.00E-06 | -1.65 | C1QB | 2.40E-01 | 1.70E-01 | 0.85 | 5.50E-02 |
| NM_000611 | CD59 | 1.00E-03 | 1.50E-04 | 0.57 | CD59 | 6.00E-01 | 9.40E-01 | -0.01 | 5.90E-02 |
| NM_000064 | C3 | 6.60E-01 | 9.30E-01 | 0.12 | C3 | 1.50E-01 | 9.50E-02 | 0.06 | 8.50E-02 |
| NM_000587 | C7 | 1.50E-01 | 6.30E-02 | -0.09 | C7 | 4.60E-01 | 5.10E-01 | -0.02 | 9.50E-02 |
| NM_001735 | C5 | 2.40E-01 | 1.20E-01 | 0.12 | C5 | 1.90E-05 | 4.00E-06 | -0.54 | 1.50E-01 |
| NM_021023 | FHR-3 | 5.10E-01 | 4.60E-01 | 0.03 | CFHR3 | 8.00E-02 | 4.20E-02 | -0.08 | 2.40E-01 |
| NM_015991 | C1QA | 4.00E-06 | <1E-16 | -2.33 | C1QA | 4.10E-01 | 4.10E-01 | 0.32 | 3.10E-01 |
| NM_030787 | FHR5 | 3.70E-01 | 2.40E-01 | -0.05 | CFHR5 | 5.60E-01 | 7.90E-01 | -0.01 | 3.10E-01 |
| NM_000574 | DAF | 3.30E-01 | 2.00E-01 | 0.14 | CD55 | 4.90E-01 | 5.90E-01 | 0.02 | 3.30E-01 |
| NM_000204 | IF | 3.60E-01 | 2.30E-01 | 0.04 | CFI | 5.90E-01 | 8.90E-01 | 0 | 3.50E-01 |
| NM_005666 | HFL3 | 4.10E-01 | 3.00E-01 | -0.06 | CFHR2 | 5.80E-01 | 8.70E-01 | -0.01 | 3.70E-01 |
| NM_007293 | C4A | 3.70E-01 | 2.50E-01 | -0.05 | C4A | 6.10E-01 | 9.80E-01 | 0 | 4.00E-01 |
| NM_007293 | C4A | 3.70E-01 | 2.50E-01 | -0.05 | C4A | 6.10E-01 | 9.80E-01 | 0 | 4.00E-01 |
| NM_007293 | C4A | 3.70E-01 | 2.50E-01 | -0.05 | C4A | 6.10E-01 | 9.80E-01 | 0 | 4.00E-01 |
| NM_007293 | C4A | 3.70E-01 | 2.50E-01 | -0.05 | C4A | 6.10E-01 | 9.80E-01 | 0 | 4.00E-01 |
| NM_182528 | LOC165257 | 5.50E-01 | 5.50E-01 | -0.04 | C1QL2 | 4.90E-01 | 5.80E-01 | -0.02 | 4.40E-01 |
| NM_006684 | FHR-4 | 4.10E-01 | 3.00E-01 | 0.05 | CFHR4 | 5.80E-01 | 8.90E-01 | 0 | 4.50E-01 |
| NM_000066 | C8B | 4.80E-01 | 4.00E-01 | 0.05 | C8B | 5.00E-01 | 6.20E-01 | 0.01 | 4.50E-01 |
| NM_000715 | C4BPA | 2.50E-01 | 1.30E-01 | 0.07 | C4BPA | 5.50E-01 | 7.80E-01 | -0.01 | 5.00E-01 |
| NM_000562 | C8A | 1.30E-01 | 4.80E-02 | 0.14 | C8A | 5.40E-01 | 7.30E-01 | -0.02 | 5.40E-01 |
| NM_016546 | C1RL | 4.60E-01 | 3.70E-01 | -0.07 | C1RL | 7.90E-02 | 4.10E-02 | 0.21 | 5.60E-01 |
| NM_000606 | C8G | 6.50E-01 | 9.10E-01 | 0.01 | C8G | 2.50E-01 | 1.90E-01 | -0.06 | 5.80E-01 |
| NM_002113 | HFL1 | 5.60E-01 | 6.00E-01 | -0.02 | CFHR1 | 2.90E-01 | 2.40E-01 | 0.05 | 6.40E-01 |
| NM_000186 | HF1 | 4.00E-01 | 2.80E-01 | -0.09 | CFH | 5.70E-01 | 8.40E-01 | 0.01 | 7.10E-01 |
| NM_201442 | C1S | 4.10E-01 | 3.00E-01 | -0.06 | C1S | 5.50E-01 | 7.60E-01 | 0.01 | 7.40E-01 |
| NM_172350 | MCP | 6.60E-01 | 9.30E-01 | -0.02 | NA | NA | NA | NA | 8.10E-01 |
| NM_006688 | CRF | 5.60E-01 | 6.00E-01 | -0.02 | C1QL1 | 4.00E-01 | 3.90E-01 | 0.03 | 8.40E-01 |
| NM_001831 | CLU | 2.90E-02 | 7.60E-03 | 0.54 | CLU | 5.00E-01 | 6.10E-01 | -0.02 | 9.00E-01 |
| NM_000065 | C6 | 4.00E-01 | 2.80E-01 | 0.04 | C6 | 3.70E-01 | 3.40E-01 | -0.03 | 9.30E-01 |

Table S10. Genes of the complement system differentially expressed between atopic and controls treated with pollen. We only show a lookup of the 8 genes that were differentially expressed in response to pollen in atopic individuals at a micro-array wide significance level (see Table S8. Complement system genetic variations associated with pollen allergic sensitisation ranked by adjusted p-value. The gene association p-value was computed with MAGENTA algorithm using SNPs within a window of 50Kbp around the gene. The best SNP with minimum p-value is reported with the corresponding FDR. Genes denoted in bold are significantly associated with pollen sensitisation (FDR < 0.05). The correlation between the best SNP p-value and # SNPs/Gene or #independent SNPs/Gene was not significant (p>0.32, r=-0.13 and r=-0.15 respectively).

|  | Gene | | | | | | Best SNP | |  |
| --- | --- | --- | --- | --- | --- | --- | --- | --- | --- |
| Symbol | adjusted | Chr | start | end | #SNPs/Gene | #Independ | Pos | p | FDR |
|  | p-value |  |  |  |  | SNPs/Gene |  |  |  |
| **C2** | 2.03E-05 | 6 | 31868775 | 31913449 | 112 | 14 | 31821040 | 1.20E-05 | 5.40E-04 |
| **C6** | 3.16E-02 | 5 | 41142335 | 41261540 | 263 | 34 | 41142605 | 6.67E-04 | 1.50E-02 |
| **CFB** | 2.44E-02 | 6 | 31913720 | 31919861 | 78 | 11 | 31915613 | 1.68E-03 | 1.80E-02 |
| **C4A** | 2.44E-02 | 6 | 31949833 | 32003194 | 94 | 14 | 31915613 | 1.68E-03 | 1.80E-02 |
| **C3AR1** | 3.90E-02 | 12 | 8210918 | 8218955 | 57 | 11 | 8208243 | 1.96E-03 | 1.80E-02 |
| **CFHR3** | 3.16E-02 | 1 | 196743929 | 196763203 | 45 | 5 | 196771124 | 3.59E-03 | 2.10E-02 |
| **CFHR1** | 3.78E-02 | 1 | 196788860 | 196801319 | 61 | 6 | 196771124 | 3.59E-03 | 2.10E-02 |
| **C9** | 1.20E-01 | 5 | 39284377 | 39364655 | 155 | 20 | 39314746 | 4.13E-03 | 2.10E-02 |
| **CLU** | 2.08E-01 | 8 | 27454450 | 27472327 | 141 | 21 | 27482869 | 4.26E-03 | 2.10E-02 |
| **CD46** | 1.30E-01 | 1 | 207925382 | 207968861 | 73 | 13 | 207980900 | 7.14E-03 | 3.00E-02 |
| **C5** | 1.41E-01 | 9 | 123714613 | 123812554 | 167 | 14 | 123835198 | 7.27E-03 | 3.00E-02 |
| **CR1L** | 2.01E-01 | 1 | 207818457 | 207897036 | 127 | 14 | 207923080 | 9.64E-03 | 3.60E-02 |
| **ITGAM** | 1.88E-01 | 16 | 31271287 | 31344213 | 71 | 11 | 31264266 | 1.14E-02 | 3.90E-02 |
| **C1QBP** | 3.83E-01 | 17 | 5336098 | 5342471 | 108 | 13 | 5308307 | 1.65E-02 | 4.80E-02 |
| **C1QA** | 3.92E-01 | 1 | 22963117 | 22966175 | 99 | 13 | 22924417 | 1.72E-02 | 4.80E-02 |
| **C1QC** | 3.75E-01 | 1 | 22970117 | 22974603 | 100 | 14 | 22924417 | 1.72E-02 | 4.80E-02 |
| ITGAX | 3.45E-01 | 16 | 31366508 | 31394318 | 62 | 13 | 31361922 | 2.22E-02 | 5.60E-02 |
| C4BPA | 4.34E-01 | 1 | 207277606 | 207318317 | 103 | 12 | 207273956 | 2.35E-02 | 5.60E-02 |
| C4BPB | 4.70E-01 | 1 | 207262211 | 207273337 | 82 | 12 | 207273956 | 2.35E-02 | 5.60E-02 |
| ITGB2 | 5.47E-01 | 21 | 46305867 | 46348753 | 134 | 19 | 46313956 | 2.98E-02 | 6.20E-02 |
| C1RL | 3.15E-01 | 12 | 7247145 | 7261869 | 52 | 6 | 7306842 | 3.04E-02 | 6.20E-02 |
| C8B | 6.07E-01 | 1 | 57394882 | 57431688 | 156 | 18 | 57469960 | 3.15E-02 | 6.20E-02 |
| C1QB | 5.17E-01 | 1 | 22979681 | 22988029 | 91 | 11 | 22952385 | 3.28E-02 | 6.20E-02 |
| CFI | 4.93E-01 | 4 | 110661847 | 110723335 | 93 | 16 | 110612536 | 3.33E-02 | 6.20E-02 |
| CFD | 5.01E-01 | 19 | 859664 | 863610 | 36 | 10 | 868114 | 4.36E-02 | 7.70E-02 |
| VTN | 5.03E-01 | 17 | 26694298 | 26697373 | 52 | 8 | 26725264 | 4.44E-02 | 7.70E-02 |
| C1S | 4.14E-01 | 12 | 7167979 | 7178335 | 28 | 7 | 7120395 | 4.62E-02 | 7.70E-02 |
| C1R | 5.16E-01 | 12 | 7187514 | 7245043 | 67 | 11 | 7162597 | 5.28E-02 | 8.50E-02 |
| C1QL2 | 7.68E-01 | 2 | 119913818 | 119916471 | 120 | 14 | 119957656 | 5.61E-02 | 8.70E-02 |
| C3 | 8.03E-01 | 19 | 6677845 | 6720662 | 121 | 30 | 6691630 | 6.01E-02 | 9.00E-02 |
| C5AR1 | 6.62E-01 | 19 | 47813103 | 47825327 | 47 | 10 | 47770937 | 6.32E-02 | 9.00E-02 |
| C1QL1 | 7.16E-01 | 17 | 43037060 | 43045644 | 72 | 14 | 42993854 | 6.38E-02 | 9.00E-02 |
| C1QL3 | 8.08E-01 | 10 | 16555741 | 16564004 | 116 | 18 | 16581628 | 6.74E-02 | 9.10E-02 |
| C7 | 8.03E-01 | 5 | 40909598 | 40983042 | 214 | 20 | 40991343 | 6.87E-02 | 9.10E-02 |
| C8A | 8.46E-01 | 1 | 57320442 | 57383894 | 141 | 28 | 57364040 | 7.16E-02 | 9.10E-02 |
| CD59 | 8.69E-01 | 11 | 33724555 | 33758025 | 171 | 27 | 33731544 | 7.29E-02 | 9.10E-02 |
| CR2 | 7.77E-01 | 1 | 207627644 | 207663240 | 106 | 11 | 207668413 | 8.63E-02 | 1.00E-01 |
| CR1 | 7.82E-01 | 1 | 207669472 | 207815110 | 165 | 15 | 207668413 | 8.63E-02 | 1.00E-01 |
| CD93 | 8.66E-01 | 20 | 23059992 | 23066977 | 153 | 12 | 23036048 | 8.88E-02 | 1.00E-01 |
| CFH | 8.37E-01 | 1 | 196621007 | 196716634 | 121 | 10 | 196580038 | 1.37E-01 | 1.50E-01 |
| C8G | 8.24E-01 | 9 | 139839697 | 139841426 | 48 | 4 | 139860630 | 1.46E-01 | 1.60E-01 |
| CD55 | 8.64E-01 | 1 | 207494816 | 207534311 | 80 | 6 | 207563312 | 1.74E-01 | 1.90E-01 |
| CFHR5 | 9.32E-01 | 1 | 196946666 | 196978803 | 83 | 12 | 197008364 | 2.19E-01 | 2.30E-01 |
| CFHR2 | 9.83E-01 | 1 | 196912933 | 196928356 | 66 | 13 | 196939189 | 3.67E-01 | 3.80E-01 |
| CFHR4 | 9.74E-01 | 1 | 196857143 | 196887843 | 82 | 10 | 196867232 | 3.75E-01 | 3.80E-01 |
| CFP | NA | 23 | 47483611 | 47489704 | NA | NA | NA | NA |  |

Table S9). We identified 5 new complement system genes specifically associated with the atopic response to pollen and confirm the importance of previously known C3AR1 and C5AR1. Significant genes depicted in bold sorted by p-value.

| Symbol | FDR  (microarray wide) | p | Log2Ratio |
| --- | --- | --- | --- |
| **C1QR1** | 7.90E-02 | 6.10E-03 | -0.92 |
| **CFP** | 1.10E-01 | 1.40E-02 | -0.18 |
| **CFD** | 1.10E-01 | 1.50E-02 | -1.07 |
| **ITGB2** | 1.30E-01 | 2.00E-02 | -0.5 |
| **ITGAX** | 1.30E-01 | 2.20E-02 | -0.37 |
| **C3AR1** | 1.50E-01 | 3.20E-02 | -0.43 |
| **C5AR1** | 1.70E-01 | 4.20E-02 | -0.74 |
| C1QBP | 3.10E-01 | 1.50E-01 | 0.17 |

Table S11. INPAR-N direct and indirect proteins connectivity is statistically significant, suggesting their involvement on common mechanism.

|  | Topological statistic | observed | expected | p-value |
| --- | --- | --- | --- | --- |
| INPAR-N | Direct Edges Count | 26 | 2.41 | 0.000999 |
|  | INPAR-N Direct Degrees Mean | 3.25 | 1.12 | 0.000999 |
|  | INPAR-N Indirect Degrees Mean | 67.9 | 33.3 | 0.000999 |
|  | CI Degrees Mean^*^ | 2.45 | 2.15 | 0.000999 |

* Average number of INPAR-N proteins that common interactors binds to.

Table S12. Disease network INPAR-N genetic variations associated with pollen allergic sensitisation ranked by adjusted p-value. The gene association p-value was computed with MAGENTA algorithm using SNPs within a window of 50Kbp around the gene. The best SNP with minimum p-value is reported with the corresponding FDR. Genes denoted in bold are significantly associated with pollen sensitisation (FDR < 0.05). The correlation between the best SNP p-value and the number of SNPs/Gene or number of independent SNPs/Gene was not significant (p>0.14, r=-0.29 and r=-0.35 respectively)

|  | Gene | | | | | | Best SNP | |  |
| --- | --- | --- | --- | --- | --- | --- | --- | --- | --- |
| Symbol | adjusted | Chr | start | end | #SNPs/Gene | #Independ | Pos | p |  |
|  | p-value |  |  |  |  | SNPs/Gene |  |  | FDR |
| **C3AR1** | 3.90E-02 | 12 | 8210918 | 8218955 | 57 | 11 | 8208243 | 1.96E-03 | 0.02 |
| **AGER** | 1.82E-01 | 6 | 32148745 | 32152023 | 174 | 19 | 32150295 | 3.27E-03 | 0.02 |
| **STAT4** | 1.45E-01 | 2 | 191894305 | 192015925 | 193 | 32 | 192011105 | 3.43E-03 | 0.02 |
| **FYN** | 2.20E-01 | 6 | 111982484 | 112194627 | 358 | 43 | 111999581 | 3.70E-03 | 0.02 |
| **CD46** | 1.30E-01 | 1 | 207925382 | 207968861 | 73 | 13 | 207980900 | 7.14E-03 | 0.03 |
| SRC | 3.70E-01 | 20 | 35973087 | 36033821 | 106 | 23 | 36051594 | 1.64E-02 | 0.05 |
| LCK | 2.16E-01 | 1 | 32716839 | 32751766 | 27 | 7 | 32729701 | 1.80E-02 | 0.05 |
| MOG | 5.80E-01 | 6 | 29624757 | 29640149 | 234 | 19 | 29575278 | 2.27E-02 | 0.05 |
| ITGA5 | 3.11E-01 | 12 | 54789044 | 54813050 | 42 | 7 | 54856135 | 2.57E-02 | 0.05 |
| ITGA3 | 4.52E-01 | 17 | 48133339 | 48167849 | 92 | 17 | 48122960 | 2.67E-02 | 0.05 |
| ITGB2 | 5.47E-01 | 21 | 46305867 | 46348753 | 134 | 19 | 46313956 | 2.98E-02 | 0.05 |
| ICAM1 | 5.23E-01 | 19 | 10381516 | 10397291 | 61 | 13 | 10338350 | 4.01E-02 | 0.06 |
| ICAM3 | 4.70E-01 | 19 | 10444451 | 10450345 | 64 | 9 | 10436561 | 4.40E-02 | 0.06 |
| PECAM1 | 4.27E-01 | 17 | 62396776 | 62407083 | 49 | 3 | 62358522 | 4.75E-02 | 0.06 |
| C3 | 8.03E-01 | 19 | 6677845 | 6720662 | 121 | 30 | 6691630 | 6.01E-02 | 0.07 |
| FN1 | 7.83E-01 | 2 | 2.16E+08 | 2.16E+08 | 199 | 22 | 216256980 | 6.28E-02 | 0.07 |
| ITGAL | 8.32E-01 | 16 | 30483982 | 30534506 | 44 | 10 | 30518095 | 1.52E-01 | 0.17 |
| CD55 | 8.64E-01 | 1 | 2.07E+08 | 2.08E+08 | 80 | 6 | 207563312 | 1.74E-01 | 0.18 |
| CD81 | 9.49E-01 | 11 | 2398546 | 2418649 | 74 | 11 | 2356768 | 2.26E-01 | 0.23 |

Table S13. Meta-analysis of the disease network (INPAR-N) genes expression showing all transcripts available in both microarrays sorted by meta-analysis p-value. Gene denoted in bold are reproducubly differentially expressed at microarray-wide significance level α<1.2×10^-6^ .

|  | Discovery | | | | Replication | | | | Meta-Analysis |
| --- | --- | --- | --- | --- | --- | --- | --- | --- | --- |
| Accession | Symbol | FDR | Pval | Log2Ratio | Symbol | FDR | Pval | Log2Ratio | p |
| mRNA RefSeq |  |  |  |  |  |  |  |  |  |
| NM_153047 | **FYN** | 3.00E-06 | <1E-16 | 1.02 | FYN | 8.90E-02 | 4.80E-02 | 0.29 | 6.60E-14 |
| NM_003151 | **STAT4** | 8.30E-04 | 1.10E-04 | 0.58 | STAT4 | 6.20E-05 | 1.30E-05 | 0.66 | 1.20E-13 |
| NM_004356 | **CD81** | 2.30E-03 | 3.80E-04 | -0.5 | CD81 | <1E-16 | <1E-16 | -0.74 | 1.40E-13 |
| NM_004054 | **C3AR1** | 9.70E-05 | 9.00E-06 | -1.26 | C3AR1 | 1.80E-05 | 3.00E-06 | -0.83 | 3.60E-13 |
| NM_000211 | **ITGB2** | 9.10E-02 | 3.10E-02 | -0.51 | ITGB2 | <1E-16 | <1E-16 | -1 | 2.10E-11 |
| NM_002037 | **FYN** | 5.00E-06 | <1E-16 | -0.95 | FYN | 2.50E-02 | 1.10E-02 | -0.28 | 4.20E-10 |
| NM_002209 | **ITGAL** | 3.10E-02 | 8.20E-03 | 0.52 | ITGAL | 1.10E-03 | 3.20E-04 | 0.35 | 8.20E-08 |
| NM_153048 | **FYN** | 4.90E-03 | 9.20E-04 | 0.6 | FYN | 1.30E-02 | 4.90E-03 | 0.4 | 1.20E-07 |
| NM_005356 | LCK | 1.50E-01 | 6.30E-02 | 0.36 | LCK | 9.20E-03 | 3.40E-03 | 0.28 | 1.80E-04 |
| NM_002205 | ITGA5 | 3.90E-02 | 1.10E-02 | -0.33 | ITGA5 | 1.10E-03 | 3.30E-04 | -0.52 | 2.40E-03 |
| NM_002433 | MOG | 2.00E-02 | 4.80E-03 | 0.14 | MOG | 2.60E-01 | 2.00E-01 | 0.04 | 4.80E-03 |
| NM_001136 | AGER | 1.10E-02 | 2.40E-03 | -0.18 | AGER | 3.50E-01 | 3.10E-01 | -0.03 | 9.70E-03 |
| NM_000064 | C3 | 6.60E-01 | 9.30E-01 | 0.12 | C3 | 1.50E-01 | 9.50E-02 | 0.06 | 8.50E-02 |
| NM_002026 | FN1 | 1.70E-01 | 7.10E-02 | -1.57 | FN1 | 5.00E-01 | 6.10E-01 | -0.05 | 2.30E-01 |
| NM_054034 | FN1 | 5.00E-01 | 4.50E-01 | 0.03 | FN1 | 2.90E-02 | 1.30E-02 | -0.13 | 3.30E-01 |
| NM_000201 | ICAM1 | 5.90E-01 | 6.90E-01 | -0.02 | ICAM1 | 3.90E-01 | 3.70E-01 | -0.04 | 3.30E-01 |
| NM_000574 | DAF | 3.30E-01 | 2.00E-01 | 0.14 | CD55 | 4.90E-01 | 5.90E-01 | 0.02 | 3.30E-01 |
| NM_005417 | SRC | 6.40E-01 | 8.70E-01 | 0.01 | SRC | 3.80E-01 | 3.70E-01 | 0.03 | 3.80E-01 |
| NM_198291 | SRC | 4.80E-05 | 4.00E-06 | -1.17 | SRC | 5.90E-01 | 9.00E-01 | 0 | 5.30E-01 |
| NM_002204 | ITGA3 | 3.10E-01 | 1.80E-01 | 0.05 | ITGA3 | 6.60E-02 | 3.40E-02 | -0.08 | 5.70E-01 |
| NM_002162 | ICAM3 | 3.80E-01 | 2.60E-01 | -0.17 | ICAM3 | 4.00E-01 | 3.90E-01 | 0.08 | 9.80E-01 |

Table S14. Biological coordination in atopic patients (cases) is disrupted in response to pollen allergen (replication dataset). In controls, the mean absolute correlation within INPAR-N and T cell activation is significantly larger in response to allergen but not in response to diluent.

|  | Allergen challenge | | | Diluent challenge | | |  |
| --- | --- | --- | --- | --- | --- | --- | --- |
|  | Mean absolute correlation | | | Mean absolute correlation | | |  |
| Gene set | Controls | Cases | P | Controls | Cases | P | N |
|  | (95% C.I.) | (95% C.I.) | (permutation) | (95% C.I.) | (95% C.I.) | (permutation) |  |
| **INPAR-N** | **0.32** | **0.27** | **4.10E-02** | 0.3 | 0.28 | 2.80E-01 | 276 |
|  | **(0.29, 0.35)** | **(0.25, 0.30)** | **-2.80E-03** | (0.27, 0.33) | (0.25, 0.30) | -1.10E-01 |  |
| INPAR-N inter-pathway | 0.28 | 0.26 | 4.10E-01 | 0.28 | 0.25 | 4.20E-01 | 145 |
|  | (0.24, 0.32) | (0.23, 0.29) | -2.90E-01 | (0.24, 0.31) | (0.22, 0.29) | -2.60E-01 |  |
| **T cell activation** | **0.28** | **0.26** | **3.20E-45** | 0.24 | 0.24 | 7.70E-01 | 57970 |
|  | **(0.28, 0.28)** | **(0.26, 0.26)** | **0.00E+00** | (0.24, 0.24) | (0.24, 0.24) | -7.30E-01 |  |
| Complement System | 0.2 | 0.25 | 5.60E-02 | 0.21 | 0.21 | 4.90E-01 | 1326 |
|  | (0.22, 0.25) | (0.24, 0.26) | -8.10E-03 | (0.20, 0.22) | (0.20, 0.22) | -4.30E-01 |  |
| Complement System vs. | 0.24 | 0.25 | 3.30E-01 | 0.22 | 0.22 | 1.20E-03 | 17727 |
| T-cell Activation | (0.24, 0.25) | (0.24, 0.25) | -2.10E-01 | (0.22, 0.22) | (0.21, 0.22) | -2.40E-04 |  |

Note: #N denotes the number of correlations in the gene set. Only correlation mean absolute differences > 0.005 and p < 0.05 are considered significant. *INPAR-N inter-pathway* only considers correlations between genes in different pathways.

Table S15. Subset of genes that interact between the complement system and T cell activation (Ingenuity® IPA)

| Symbol | Entrez Gene Name | Location | Family | Drugs |
| --- | --- | --- | --- | --- |
| AGER | advanced glycosylation end product-specific receptor | Plasma Membrane | transmembrane receptor |  |
| CD46 | CD46 molecule, complement regulatory protein | Plasma Membrane | other |  |
| CD55 | CD55 molecule, decay accelerating factor for complement (Cromer blood group) | Plasma Membrane | other |  |
| CD81 | CD81 molecule | Plasma Membrane | other |  |
| C3 | complement component 3 | Extracellular Space | peptidase |  |
| C3AR1 | complement component 3a receptor 1 | Plasma Membrane | G-protein coupled receptor |  |
| FN1 | fibronectin 1 | Plasma Membrane | enzyme |  |
| FYN | FYN oncogene related to SRC, FGR, YES | Plasma Membrane | kinase | Dasatinib |
| ITGA3 | integrin, alpha 3 (antigen CD49C, alpha 3 subunit of VLA-3 receptor) | Plasma Membrane | other |  |
| ITGA5 | integrin, alpha 5 (fibronectin receptor, alpha polypeptide) | Plasma Membrane | other |  |
| ITGAL | integrin, alpha L (antigen CD11A (p180), lymphocyte function-associated antigen 1; alpha polypeptide) | Plasma Membrane | other | Efalizumab |
| ITGB2 | integrin, beta 2 (complement component 3 receptor 3 and 4 subunit) | Plasma Membrane | other |  |
| ICAM1 | intercellular adhesion molecule 1 | Plasma Membrane | transmembrane receptor |  |
| ICAM3 | intercellular adhesion molecule 3 | Plasma Membrane | other |  |
| LCK | lymphocyte-specific protein tyrosine kinase | Cytoplasm | kinase | Dasatinib, pazopanib |
| MOG | myelinoligodendrocyte glycoprotein | Plasma Membrane | other |  |
| PECAM1 | platelet/endothelial cell adhesion molecule | Plasma Membrane | other |  |
| STAT4 | signal transducer and activator of transcription 4 | Nucleus | transcription regulator |  |
| SRC | v-src sarcoma (Schmidt-Ruppin A-2) viral oncogene homolog (avian) | Cytoplasm | kinase | Dasatinib, saracatinib,  AZM-475271 |

# Methods S1

## Pathway enrichment for genetic variation associated with clinical phenotypes

The pathways identified (complement system, INPAR-N and T cell activation) were re-analysed to assess and reproduce their enrichment for SNPs statistically associated with (α=10^-2^) grass pollen sensitisation using our approach [[1](#_ENREF_1)]. A SNP is considered to tag a gene if it lies within a conservative 5 Kbp window around the gene. We used the hypergeometric distribution to compute the probability of the pointwise probability of sampling $\geq k$ SNPs in any of the pathways considered. The probability that the number of SNPs found in a given pathway occurred by chance is the cumulative probability of finding $k$ or more SNPs in the gene subset. The effect size is the odds ratio contrasting between the proportion of significant SNPs among SNPs within the pathway as compared with the same proportion of significant SNPs in the remaining genes for non-pathway SNPs.

## Genetic variations associated with pollen allergic sensitisation within INPAR-N genes

Genetic variations within 50 Kbp around complement system genes were investigated for SNP associated with pollen allergic sensitisation. The minimum and adjusted p-values were computed using magenta that corrects for multiple confounders in genome wide association studies. SNPs significantly associated (FDR < 0.05) were highlighted.

## Statistical sensitivity analysis of the complement system enrichment

A sensitivity analysis of the complement system enrichment was conducted to assess the dependency of the results on the fold change and on the false discovery rate cut-offs that jointly determine if a gene is differentially expressed. The odds ratio of the enrichment was assessed for a cut-off of the fold expression change range from (1-5). This analysis was independently repeated for a wide range of cut-offs applied to the FDR (0.01-0.1). Number of differentially expressed genes was recorded for all FDR and fold change cut-off combinations to assess the robustness of effect on the pathway enrichments. We also performed a similar sensitivity analysis to the enrichment for genetic associations with grass pollen sensitisation. We tested several windows around the genes up to 500Kb as used by i-GSEA and increasing SNP significance cut-off (α=0.001).

## Gene expression microarray pre-processing

Gene expression arrays were normalised with quantile normalisation [[2](#_ENREF_2),[3](#_ENREF_3)]. The normalized intensities of the initial probes were then filtered out to remove probes with low absolute values, low profile variance and detection score below 0.80. Overall, 10% of the probes were filtered out (4734), and 42559 were kept on the dataset for further analysis.

## Differential Expression Analysis

A two-sample permutation test based on the Welch t-statistic with 10^4^ permutations was used. The False Discovery Rate (FDR) was set below 1% using Storey’s algorithm. A volcano plot analysis filtered genes with low fold change ratio (absolute ratio< 4). Interactome mapping was conducted to identify a subnetwork enriched for differentially expressed genes using Bionet [[4](#_ENREF_4)].

# References

1. Barrenas F, Chavali S, Couto Alves A, Coin L, Jarvelin M-R, et al. (2012) Highly interconnected genes in disease-specific networks are enriched for disease-associated polymorphisms. Genome Biology 13: R46.

2. Bolstad BM, Irizarry RA, Åstrand M, Speed TP (2003) A comparison of normalization methods for high density oligonucleotide array data based on variance and bias. Bioinformatics 19: 185-193.

3. Irizarry RA, Hobbs B, Collin F, Beazer‐Barclay YD, Antonellis KJ, et al. (2003) Exploration, normalization, and summaries of high density oligonucleotide array probe level data. Biostatistics 4: 249-264.

4. Beisser D, Klau GW, Dandekar T, Müller T, Dittrich MT (2010) BioNet: an R-Package for the functional analysis of biological networks. Bioinformatics 26: 1129-1130.
